# Recent advances in understanding adverse effects associated with drugs targeting the serotonin receptor, 5-HT GPCR

**DOI:** 10.3389/fgwh.2022.1012463

**Published:** 2022-12-08

**Authors:** Isabella Marie Jamu, Haruko Okamoto

**Affiliations:** Department of Biochemistry & Biomedicine, the School of Life Sciences, University of Sussex, Brighton, United Kingdom

**Keywords:** serotonin 5-HT, GPCR (G protein coupled receptor), CNS—central nervous system, PNS—peripheral nervous system, tryptophane hydroxyrase (TPH), oestrogen (E) 2, prolactin

## Abstract

It has been acknowledged that more women suffer from adverse effects of drugs than men globally. A group of drugs targeting serotonin [5-hydroxytryptamine] (5-HT) binding G-protein-coupled receptors (GPCRs) have been reported to preferentially affect women more than men, causing adverse effects such as breast cancer and infertility. 5-HT GPCR-targeted drugs in the central nervous system (CNS) manage psychiatric conditions, such as depression or bipolar and in the peripheral nervous system (PNS) treat migraines. Physiological characteristics such as specific types of hormones, higher body fat density and smaller body mass in women result in disparities in pharmacodynamics of drugs, thus explaining sex-related differences in the observed adverse effects. In this review, we discuss the side effects of drugs targeting 5-HT GPCRs based on serotonin's roles in the CNS and PNS. We have systematically reviewed adverse effects of drugs targeting 5-HT GPCR using information from the Food and Drug Administration and European Medicines Agency. Further information on drug side effects and receptor targets was acquired from the SIDER and DrugBank databases, respectively. These drugs bind to 5-HT GPCRs in the CNS, namely the brain, and PNS such as breasts, ovaries and testes, potentially causing side effects within these areas. Oestrogen affects both the biosynthesis of 5-HT and the densities of 5-HT GPCRs in given tissues and cells. 5-HT GPCR-targeting drugs perturb this process. This is likely a reason why women are experiencing more adverse effects than men due to their periodic increase and the relatively high concentrations of oestrogen in women and, thus a greater incidence of the oestrogen-mediated 5-HT system interference. In addition, women have a lower concentration of serotonin relative to men and also have a relatively faster rate of serotonin metabolism which might be contributing to the former. We discuss potential approaches that could mitigate at least some of the adverse effects experienced by women taking the 5-HT GPCR-targeting drugs.

## Introduction

Serotonin [5-hydroxytryptamine] (5-HT) receptors belong to the rhodopsin class A family of G-protein-coupled receptors (GPCRs) (1) and are implicated in processes such as aggression, emesis, mood, and cognition (2, 3). There are fourteen 5-HT-binding receptors encoded by the human genome, of which one is an ion channel and the remaining thirteen are GPCRs. The GPCRs are grouped into six subtypes (5-HT1, 5-HT2, 5-HT4, 5-HT5, 5-HT6 and 5-HT7,receptors) based on their function and structure. 5-HT2, 5-HT4, 5-HT6 and 5-HT7 are excitatory receptors, while 5-HT1 and 5-HT5 receptors are inhibitory (4, 5). 5-HT1, 5-HT2, and 5-HT5 subtypes have further isoforms, such as 5-HT1a, 5-HT1b, 5-HT1d, 5-HT1e, 5-HT1f, 5-HT2a, 5-HT2b, 5-HT2c, 5-HT5a, and 5-HT5b. 5-HT5b is a pseudogene in humans (Table 1). 5-HT GPCRs are present in both the central nervous system (CNS) and peripheral nervous system (PNS) and have a distinct short N-terminal extracellular domain that binds to serotonin to induce a cellular response (1).

Serotonin is a type of catechol amine that serves as a neurotransmitter in the CNS as well as a hormone, regulating many fertility- and postnatal-morphogenesis of mammary glands, important for reproduction. Serotonin circulates in the cardiovascular system mostly stored in platelets (2). In the gut, serotonin is synthesised from an essential amino acid, tryptophan by tryptophan hydroxylase (TPH), which produces 5-hydroxytryptophan. Aromatic *l*-amino acid decarboxylase converts 5-hydroxytryptophan into serotonin (5, 6). It has been reported that women metabolise serotonin at a greater rate than men (7). In addition, men have a higher rate of serotonin synthesis than women (8). On average, serotonin levels are generally found to be higher in men than in women by approximately 50%. Moreover, there is a change in serotonin concentration over the 28-day periodic cycle in women (7), which poses a serious challenge in drug administration.

5-HT GPCR-targeting drugs generally have a complementary structure to their target receptor binding sites found in the CNS and PNS. 5-HT GPCR-targeting drugs act as agonists or antagonists. Excitatory-type 5-HT GPCRs are activated by agonists and inhibited by antagonists. Inhibitory-type 5-HT GPCRs inhibit a given pathway when bound to 5-HT in a negative feedback loop and therefore, agonists will replace such activity by 5-HT in promoting negative feedback, while antagonists will block the inhibitory pathway. 5-HT GPCR-targeting drugs generally have a complementary structure to its target receptor binding sites found in the CNS and PNS, where antagonists intended to block excitatory-type 5-HT GPCRs, could also bind to inhibitory-type 5-HT GPCRs thereby potentially competing with 5-HT and prevent the normal inhibitory pathway function. Similarly, agonists that activate excitatory-type 5-HT GPCRs may activate the inhibitory-type 5-HT GPCRs that further enhance the inhibitory pathway. When the intended target is an inhibitory 5-HT GPCR, drug treatment could also affect the excitatory-type 5-HT GPCRs. In both cases, excess activation of excitatory and inhibitory type 5-HT GPCRs could result in adverse effects (9).

The pharmaceuticals are intended to elicit their desired response in the PNS or CNS. However, there have been a few reports indicating that those remaining in PNS perturb the normal function of 5-HT GPCRs in systems such as serotonin-regulated milk production in breasts, muscle contraction in ovaries and sperm production in testis causing adverse effects within these organs (10). Side effects reported amongst those drugs targeting 5-HT GPCRs include libido decrease/increase, headache, abdominal pain and/or infertility (Table 2 and Supplementary Table S2). Some 5-HT GPCR-targeted drugs cause severe adverse effects, such as hyperprolactinemia, which is also seen more often in women than men (11).

Current drug trial regimes test primarily men throughout the clinical trial phases. A review in 2018 reported that 72% of randomized controlled trials testing efficacy of drugs did not describe sex specific analysis (12). Despite the data being available, many of the studies are not reporting gender specific data. In addition, medics and researchers across the world have suggested that more women should be involved in clinical trials to evaluate and adjust the dosage and timing of drug administration for women (10, 12). Moreover, hormonal cycles and their dynamic changes at the time of any drug administration are the factors that influence sex differences' side effects (10). Clearly, a great deal of consideration is still required to understand how sexual hormones, such as oestrogen, androgen and testosterone, mediate sex-based differences in pharmacodynamics.

In this review, we will discuss the propensity of neurological pharmaceuticals targeting 5-HT GPCR to interfere with the normal function of serotonin in the CNS and PNS. The roles of sexual hormones, oestrogen, androgen and testosterone in the 5-HT system were analysed to understand how targeting 5-HT GPCRs could perturb or hinder the regulatory effect of sexual hormones on 5-HT biosynthesis alter the expression of 5-HT GPCRs that potentially contribute to side effects. Furthermore, we will discuss how the internal physiological system in women is more vulnerable to neurological pharmaceuticals, which increases the chance of side effects (Supplementary Table S1).

### Serotonin in the CNS and PNS

According to Human Protein Atlas (https://www.proteinatlas.org), all thirteen 5-HT GPCRs are expressed in the human brain (13). In the CNS, serotonin is produced in the cell bodies of serotonergic neurons found in the raphe nuclei from where serotonin is stored and released. These serotonergic neurons send signals toward ascending projections that terminate in the midbrain, limbic, cortical and hindbrain regions in a defined manner (14). Serotonin binds to 5-HT GPCRs in all regions of the brain. Individual neurons may express more than one type of 5-HT GPCR and serotonergic receptors can function on their own or in concert with other subtypes. For example, 5-HT2c and 5-HT1a receptors together modulate anxiety, while 5-HT2c receptor expressed on its own mediate appetite, locomotion and reward in neuronal cells.

Dysfunction of the serotonergic system is implicated in the pathogenesis of neurological and psychiatric disorders (14). Examples of disorders treated by 5-HT GPCR-targeted drugs include schizophrenia, generalized anxiety disorder (GAD), obsessive-compulsive disorder (OCD), major depressive disorder (MDD), premenstrual dysphoric disorder (PMDD), migraine, dravet syndrome, anti-heart burn and menopausal symptoms (Table 2). The average age of onset of psychiatric disorders on the above list lies between 18 and 25. It is widely accepted that the average age of the onset of GAD and OCD is approximately 30 years old, menopause is usually between 42 and 52 years of age, whereas that of dravet syndrome is 1–18 months (15–17). Elevated levels of serotonin in the brain could result in schizophrenia. Thus, treatment methods for this condition is to compensate for the hyper activities of both inhibitory and excitatory 5-HT GPCRs (Table 2). Conversely, GAD, OCD, MDD, PMDD, migraine, dravet syndrome and menopausal symptoms are caused by reduced serotonin levels in the brain. These disorders require mitigation of reduced activities of excitatory and inhibitory 5-HT GPCRs (Table 2). Bipolar patients can experience either an elevation or decrease in serotonin depending on whether they are experiencing a manic or depressive episode, respectively (18).

Serotonin has been shown to increase prolactin, a peptide hormone synthesized by lactotrophs in the anterior pituitary and is essential for lactation, breast development, pregnancy, sexual intercourse, ovarian functioning, regulation of progesterone-secreting structures and inhibition of hypothalamic gonadotropin-releasing hormone (19). Prolactin concentration is generally lower in men compared to women and it is at the highest in women during pregnancy (20). In mammals, intravenous administration of serotonin results in increased prolactin secretion (19, 21). Serotonin induces the release of prolactin releasing factors (PRFs), such as oxytocin and vasoactive intestinal peptide from the hypothalamus. PRFs influence lactotrophs in the anterior pituitary gland to release prolactin (19, 21).

Serotonin can cause vasoconstriction or vasodilation through either inhibitory (5-HT1) or excitatory (5-HT2) 5-HT GPCRs, respectively (22). In the extracerebral carotid arteries, serotonin binds to serotonergic receptors (5-HT1b and 5-HT1d) causing contraction of vascular smooth muscle cells and vasoconstriction of blood vessels. Migraines occur when there is a reduction in serotonin that leads to vasodilation of intracranial extracerebral blood vessels. When blood vessels dilate, the trigeminovascular system triggers the release of vasodilators, such as calcitonin gene-related peptide (CGRP), which further exacerbates pain responses. The reduction of oestrogen during menstruation and the late-luteal phase of the menstrual cycle can in-turn reduce serotonin concentration in the cardiovascular system and is potentially a cause of migraines in some women during their menstrual cycle (23).

### Synergistic and antagonistic effect of sexual hormones and serotonin in the brain

**Figure 1:**
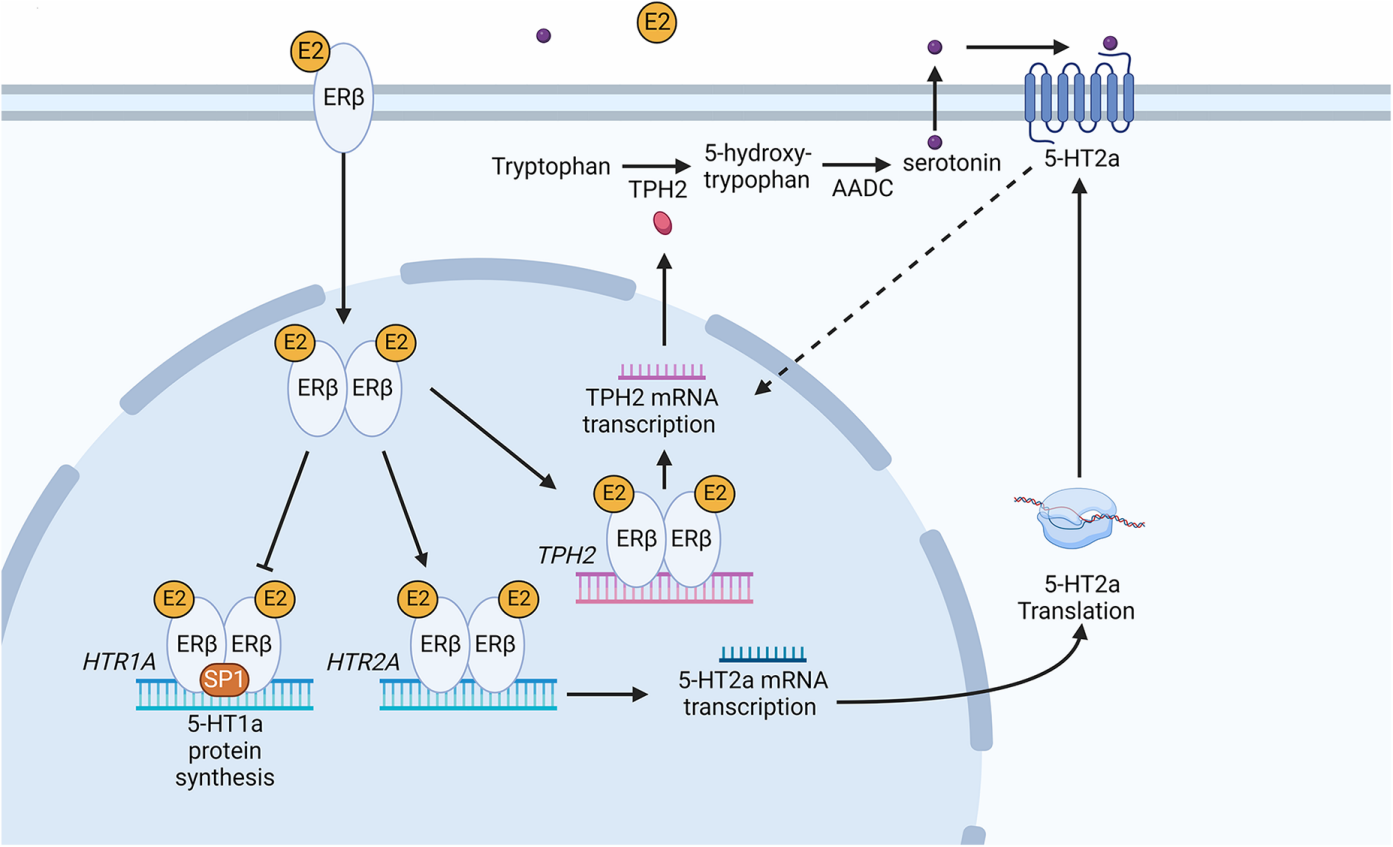
Schematic diagram of the serotonin synthesis pathway following an increase in oestradiol in the peripheral nervous tissue. The increase in E2 leads to the activation of ERβ causing it to dimerize. Dimerized ERβ interacts with SP1 to inhibit *HTR1A* receptor transcription. Secondly, ERβ causes transcription of the *HTR2A* receptor gene, increasing 5-HT2a receptor protein in the cell. An increase in 5-HT2a results in an increase in *tryptophan hydroxylase 2 (TPH2)* mRNA and subsequently TPH2 protein. The dotted line represents an indirect induction of *TPH2* by 5HT2a. Dimerized ERβ directly induces *TPH2* transcription leading to TPH2 protein synthesis. TPH2 catalyses the conversion of tryptophan into 5-hydroxytryptophan, which is then converted into serotonin (5-hydroxytryptamine) *via* aromatic *l*-amino acid decarboxylase (AADC). Serotonin binding with 5-HT2a leads to positive feedback increasing TPH2 and resulting in serotonin production. Created with BioRender.com.

There is evidence of significant interactions between sexual hormones and serotonin biosynthesis enzymes such as TPH in the CNS. There are 2 forms of TPH encoded by the human genome. TPH1 is localised both in the brain and body, whereas TPH2 is primarily expressed in the brain (13). Studies using primates and mammals showed there is a positive correlation between the accumulation of oestradiol and progesterone and the TPH enzyme (24). Results using oestrogen receptor (ER)α and ERβ KO mice showed that ERβ transcription factor is translocated to the nucleus and binds to the *TPH1* promoter when oestrogen levels increase and this leads to an accumulation of TPH1 (25). In an *in vitro* system in the presence of oestrogen, ERβ was also shown to upregulate TPH2 by binding to the oestrogen response element in the promoter (Figure 1) (26).

**Figure 2:**
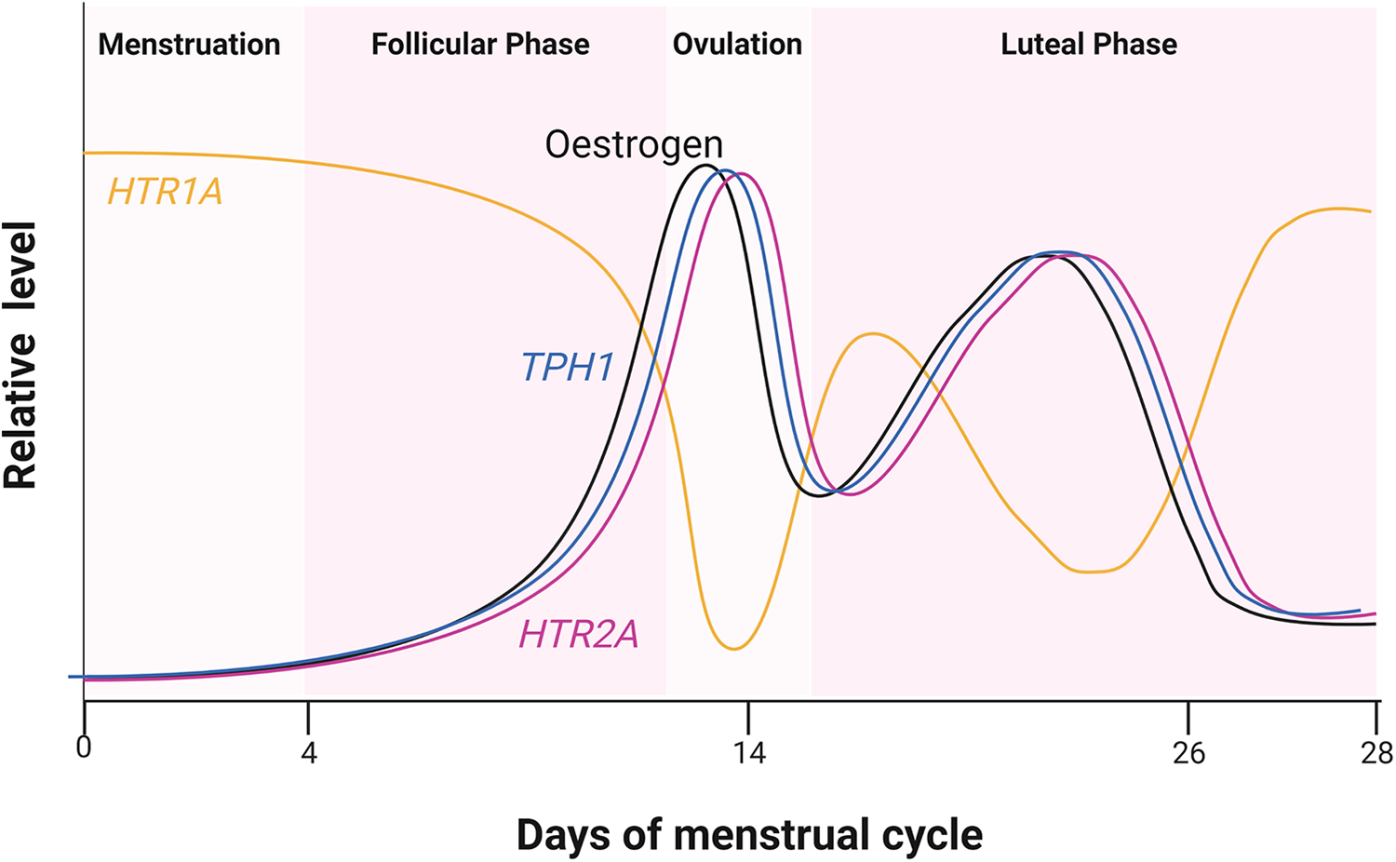
Relative hormone level during the four estrus phases. Cycling of oestrogen levels during the menstrual cycle was compared to the expression of *HTR2A* and *HTR1A* encoding 5-HT2a and 5-HT1a receptors, respectively. Oestrogen receptor (ER) also regulates *TPH1* encoding one of the serotonin biosynthesis genes. Created with BioRender.com.

Sexual hormones were also shown to regulate serotonin receptor isoforms 5-HT2a and 5-HT1a. In the case of 5-HT2a receptor, the effect of female hormones was synergistic (27), whereas the hormones act antagonistically to the accumulation of 5-HT1a receptors (Figure 2). Studies of post-menopausal women between the ages 45–55, receiving 17β-oestradiol (E2) hormonal replacement therapy for 14 weeks, followed by a maximum of 6 weeks of E2 and micronized progesterone have shown there is a positive correlation between an increase in oestrogen level and serotonin binding to the 5-HT2a receptor (28). PET scanning of the frontal cortex, to visualize [18F] altanserin labelled 5-HT2a receptor, indicated that in the presence of E2 and progesterone, 5-HT2a receptor expression increases (28). An increase in oestrogen concentration in the hypothalamus of rhesus macaque monkeys caused a decrease in radiolabelled [3H]-8-OH-DPAT binding to the 5-HT1a receptor and *HTR1A* gene expression (29). Safe (2001) has shown in an *in vitro* system that an increase in E2 initiated a decrease in *HTR1A* expression by causing the trimer complex formation of the transcription factor, specificity protein 1 (Sp1), and homodimer ERβ. The trimer complex is translocated to the nucleus to inhibit *HTR1A* transcription *via* binding to the ER binding element region of *HTR1A* (Figure 1). It was proposed that the binding of these transcription factors recruit unknown proteins and repress *HTR1A* expression when oestrogen concentrations increase (30). Conversely, *HTR1A* expression increases when oestrogen decreases. A study, using COS-1 cells transiently co-transfected with *HTR1A* promoter fused to a reporter gene and an expression vector encoding ERα or ERβ, suggested that activation of ERα results in *HTR1A* expression. The AF-1 domain specific to ERα receptor allows it to interact with the nuclear factor kappa-light-chain-enhancer of activated B cells (NF-kB) and this interaction is essential for the activation of *HTR1A* promoter (31).

These results clearly indicated that oestrogen plays a major role in 5-HT1a and 5-HT2a receptor regulation in women. The following studies showed that the small amount of oestrogen in men also plays a role in 5-HT2 receptor expression. Autoradiography showed that radiolabelled 5-HT2a receptor density was reduced in the frontal, piriform and cingulate cortex, the nucleus accumbens and olfactory tubercle of male Wistar rats upon castration (32). An increase in the number of radio-labelled 5-HT2a receptors was observed only when oestradiol benzoate (EB) or testosterone propionate (TP) were present and not with dihydrotestosterone (DHT). It was speculated that this increase in 5-HT2a receptor density was due to an endogenous aromatase converting TP to E2. The authors concluded that only oestrogen converted from testosterone *in vivo* increases 5-HT2a receptor mRNA accumulation and 5-HT2a GPCR protein levels (32).

### Serotonin and serotonergic GPCRs in women

All 13 serotonergic GPCRs are also present in the PNS. However, the localization of each is not identical between women and men (Table 1). Human Protein Atlas shows that 5-HT1, 5-HT2, 5-HT5 and 5-HT7 receptor mRNA and protein are present in the breasts of women but they are not detected in that of men (13). Serotonin is known to be synthesised and secreted from the mammary glands in female breasts (6). The development of mammary glands and milk production in humans requires a concerted sequence of events. Prolactin released from the pituitary gland in the CNS induces serotonin production in the breast tissue. There is a negative feedback loop between prolactin and serotonin. Prolactin promotes milk production while serotonin inhibits it in humans. Matsuda et al. (2004) and Stull et al. (2007) showed in mice that prolactin stimulates the expression of serotonin biosynthesis genes, *TPH1* and *aromatic l-amino acid decarboxylase.* Both genes were detected in the breast tissues at many developmental stages, particularly during nulliparous, milk filling, pregnancy, postpartum and involuting. Breastfeeding triggers a reflex secretion of prolactin from the pituitary gland in the CNS and the expression of serotonin biosynthesis genes forms part of a negative-feedback system, induced in response to stimulation by prolactin during milk filling (6). Histological analysis of mammary gland cultures of pregnant mice showed the addition of serotonin in the presence of prolactin results in the collapse of alveolar clusters (6). It has been shown that binding of serotonin to the 5-HT7 receptor leads to a decline in key scaffolding proteins forming tight junctions between epithelia resulting in the leakage of lactose to the basolateral side of the cell that represses milk production from the epithelial cells (33).

**Table 1:** Serotonin receptor expression in sex organs. GPCR 5-HT receptors expressed in female and Male organs according to Human Protein Atlas (13).

GPCR serotonin receptor	Female organs	Male organs
5-HT1a	Ovary, breasts	Testis, seminal vesicle
5-HT1b	Placenta, vagina, breasts, cervix, endometrium, fallopian tube, ovary	Testis, seminal vesicle, prostate
5-HT1d	Fallopian tube, endometrium, breasts, placenta	Testis, prostate, seminal vesicle
5-HT1e	Placenta, vagina, breasts, cervix, endometrium, fallopian tube, ovary	Testis, epididymis, prostate, seminal vesicle
5-HT1f	Placenta, vagina, breasts, cervix, endometrium, fallopian tube, ovary	Testis, epididymis, prostate, seminal vesicle
5-HT2a	Vagina, breasts, cervix, endometrium, fallopian tube, ovary	Testis, prostate, seminal vesicle
5-HT2b	Placenta, vagina, breasts, cervix, endometrium, fallopian tube, ovary	Testis, prostate, seminal vesicle
5-HT2c	None reported	None reported
5-HT4	Cervix, endometrium, fallopian tube	None reported
5-HT5a	Breast, endometrium, fallopian tube, placenta	Testis, epididymis, seminal vesicle
5-HT5b	Pseudogene	Pseudogene
5-HT6	None reported	Testis
5-HT7	Vagina, breasts, cervix, endometrium, fallopian tube, ovary	Testis, epididymis, prostate, seminal vesicle

**Figure 3:**
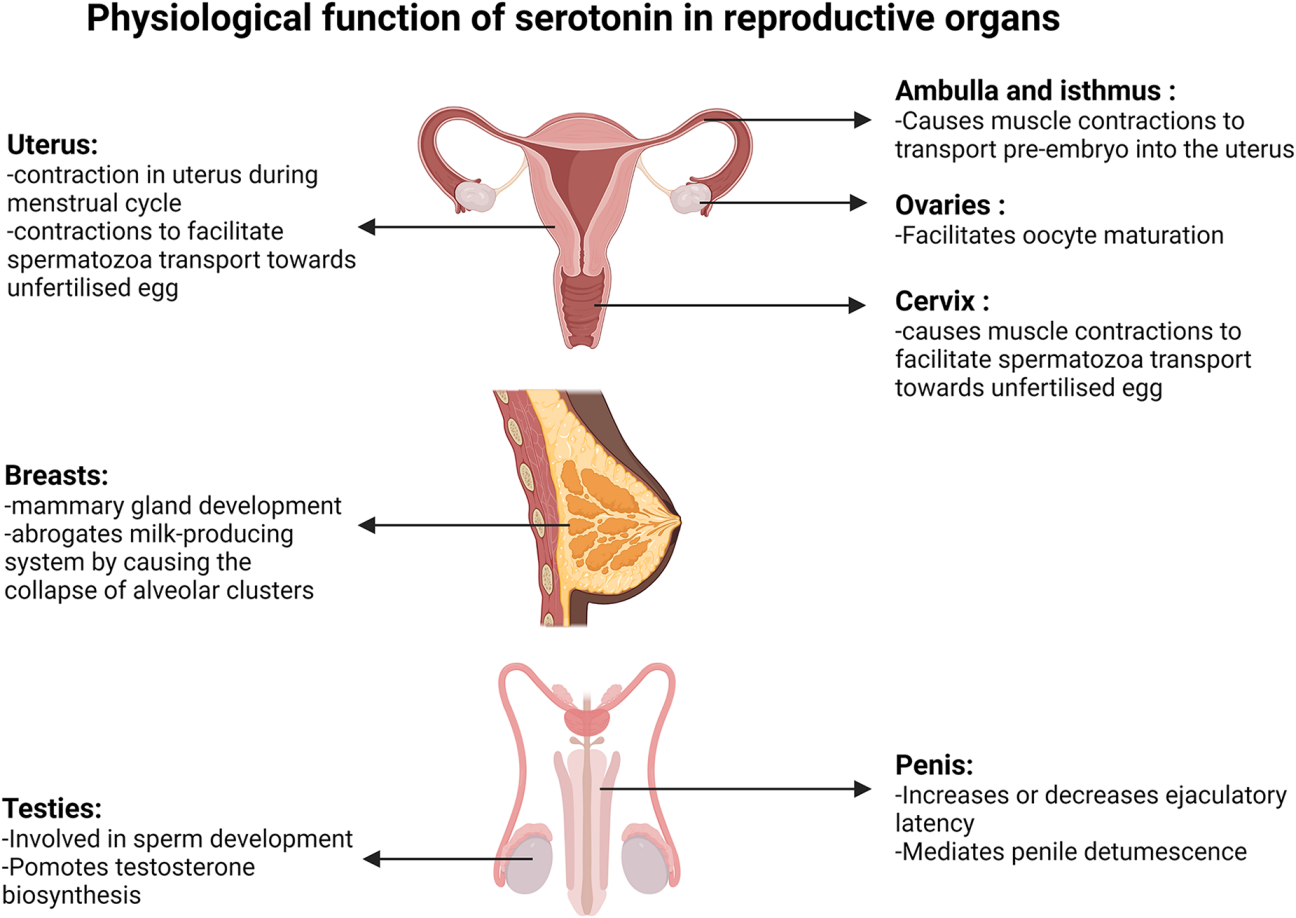
Physiological function of serotonin in reproductive organs. Diagrams present the function of serotonin in the uterus, fallopian tube, ovaries, cervix, breasts, testis and penis. Created with BioRender.com.

Studies also showed that while prolactin promotes it, serotonin suppresses expression of the *β-casein* gene that encodes a milk protein found in mammalian milk, which is a bioactive peptide essential for suckling by infants (34). Western blot analysis indicated that with elevated levels of prolactin, there is an increase in *β-casein* mRNA in MCF-12A human mammary epithelial cell (35, 36). Prolactin binds to the receptors found in the mammary epithelial cell, which triggers activation and transcription of *β-casein*. An increase in locally produced serotonin in the mammary gland has been shown to decrease β-Casein in a concentration-dependent manner. It was concluded that serotonin attenuates β-Casein accumulation (6, 36). Taken together, breast tissues receive autocrine as well as paracrine serotonergic signals essential for the maintenance of milk homeostasis (Figure 3).

Serotonin and oestrogen act synergistically in uterus and cervix contractions. Oestrogen concentration is at its lowest during menstruation, then gradually increases during the follicular phase that culminates between days 12 and 14 and peaks again during the mid to early luteal phase (Figure 2). Reports from a non-invasive ultrasound study in women revealed that the frequency of uterine contraction increases during the late follicular phase and declines throughout the luteal phase (37). Additionally, serotonin was shown to enhance uterine and cervix peristaltic contraction during the early phases of the menstrual cycle when oestrogen is elevated (38). The absence of uterine contractions may lead to endometriosis, dysmenorrhea or prevention of oocyte migration from the ovaries to the uterus, which all potentially lead to infertility (37). It has been demonstrated that serotonin enhances the contractions initiated by oestrogen during the migration of sperm towards the oviduct, likely contributing to the chances of egg fertilization (Figure 3). Hysterosalpingoscintigraphy, intrauterine pressure and oestrogen measurement showed that, by imitating sperm entrance into the posterior vaginal fornix, oestradiol concentration and thereby uterine contractions increase (39). It was also shown that E2 perfusion causes uterine peristalsis with a cervico-fundal direction (40). Oestrogen and serotonin synergistically mediate contractions in the uterus and cervix (38) and elevated levels of serotonin and 5-HT2a receptors were postulated to influence uterine contractions (41). 5-HT2 receptors coupling to the Gαq-protein family, stimulated by serotonin, have been shown to cause activation of the phospholipase C/intracellular calcium pathway, ultimately resulting in uterine muscle contraction in rats (42). Oviduct motility is important for pre-embryo and gamete transportation (Figure 3). Radioligand binding assay showed that serotonin binding to 5-HT7 receptors in muscles of the isthmus and ampulla, found in the oviduct, caused muscle relaxation (43). Serotonin has been shown to bind to 5-HT2a, 5-HT2b and 5-HT7 receptors in the ovaries and through receptor-mediated actions on Ca^2+^ and cAMP, control mammalian follicular growth and oocyte meiotic maturation (44).

### Serotonin and serotonergic GPCRs in men

Effects of serotonin on fertility are not confined to women and there is evidence that it also influences male reproductive performance. In male testicles, serotonin is involved in normal sperm development and testosterone biosynthesis (Figure 3). Inhibiting serotonin synthesis causes a decrease in follicle-stimulating hormone (FSH) and testosterone concentration in blood samples. Using a membrane integrity assay, caspase activity measurement, and flow cytometry tests it was shown that in the absence of serotonin, the number of viable sperm decreases, the sperm membrane integrity declines and there is an increase in abnormal sperm production (45).

Serotonin binding to 5-HT GPCRs can increase or decrease ejaculatory latency (Figure 3). Intraurethral infusion of serotonin showed that serotonin decreases the threshold urethral perfusion pressure and increases the number of ejaculation reflexes (46). Studies in rats and rhesus monkeys showed that 5-HT1c and 5-HT1d receptor agonists inhibit ejaculation in a dose dependent manner. Conversely, serotonin facilitates male ejaculation by interacting with 5-HT1a and 5-HT2 receptors, while administration of 5-HT2 receptor antagonists inhibits male ejaculation in mice (46, 47).

### Side effect of drugs targeting serotonin 5-HT GPCRs

Studies have shown that 40% of men and 60% of women treated with antipsychotics had a prolactin level above the upper limit of the normal range (15–25 µg/L) (11). Hyperprolactinemia is unlikely a symptom of psychiatric disorders as the level of prolactin found in healthy adults and patients with psychiatric conditions prior to drug administration are comparable (11). It is suggested that neurological drugs targeting 5-HT GPCRs in the pituitary gland increase prolactin in both women and men often to above the normal levels and is therefore, one of the most common side effects caused by those drugs. Drugs that are agonists to excitatory-type 5-HT GPCR, such as 5-HT2a, mimic serotonin, which results in prolactin secretion from lactotroph cells in breast tissue into the bloodstream. Antagonist drugs to inhibitory-type 5-HT GPCR, for example 5-HT1a, remove prolactin inhibitory pathways and thus prolactin levels remain elevated in the bloodstream (48). The 5-HT GPCR antagonists risperidone, olanzapine and paliperidone, and the agonist ziprasidone (Table 2) are all reported to cause hyperprolactinemia. Side effects linked to hyperprolactinemia are decreased fertility, headaches, reduced spermatogenesis, galactorrhoea, amenorrhea and androgen deficiency, leading to erectile dysfunction and decrease in libido (60, 61). Hyperprolactinemia, caused by antipsychotics, may be further exacerbated in women as the result of a higher baseline prolactin level compared to that of men. Pregnant women may experience hyperprolactinemia from even higher levels of prolactin secreted by the immune system, uterus, placenta and mammary glands compared to those who are not pregnant (20). Serotonin interacts with 5-HT GPCRs, triggering prolactin synthesis in the breast tissue. Consequently, breast development, lactation and pain may occur in breasts (21). Drugs such as trazodone hydrochloride and citalopram hydrobromide cause lactation, breast enlargement and pain (Supplementary Table S2) and it is possible that these drugs mimic serotonin to cause these effects.

**Table 2:** Drugs used to treat psychiatric and neurological conditions that target serotonin receptors and their side effects in women and men. Data obtained from EMA and FDA drug approving bodies, SIDER and DrugBank. Abbreviations: MDD, multiple personality disorder; OCD, obsessive compulsive disorder; GAD, generalized anxiety disorder; PMDD, premenstrual dysphoric disorder; SAD, social anxiety disorder; PD, panic disorder; 5-HT, serotonin. Orange coloured cells represent excitatory receptors and blue represents inhibitory receptors.

Generic Drug Name	Treatment	Side effect seen more in women than in men	Side effect seen more in men than in women	5-HT receptor Target	Antagonist/Agonist	References
Cisapride	Anti-heart burn	Fatal heart disturbance, vaginitis, reproductive failure, galactorrhoea	Gynecomastia	5-HT2a	Agonist	(49, 50)
				5-HT2c		
				5-HT4		
Cyproheptadine Hydrochloride	Hypersensitivity Reactions symptoms	None reported	None reported	5-HT2a	Antagonist	(49–51)
				5-HT2b		
				5-HT2c		
				5-HT7		
Fenfluramine Hydrochloride	Dravet Syndrome	None reported	None reported	5-HT1a	Agonist	(50, 52)
				5-HT1d		
				5-HT2a		
				5-HT2b		
				5-HT2c		
Amisulpride	Schizophrenia, PONV	Vaginal inflammation, menstrual disorder, pregnancy risk increase, infertility	Ejaculatory dysfunction	5-HT2a	Antagonist	(50, 52–54)
				5-HT2b		
				5-HT7		
Aripiprazole Mylan	Schizophrenia, Bipolar I	Decreased libido, tardive dyskinesia (older women), weight gain	Erectile dysfunction, dystonia	5-HT1a	*P*artial agonist	(49, 50, 52, 55)
				5-HT1b	Antagonist	
				5-HT1d		
				5-HT2a		
				5-HT2c	Antagonist, partial agonist	
				5-HT7		
Cariprazine Hydrochloride	Schizophrenia, Bipolar	Tardive dyskinesia (older women), decrease fertility, amenorrhea, vaginitis	Ejaculation dysfunction	5-HT1a	Partial agonist	(18, 49, 50)
				5-HT2a	Antagonist	
				5-HT2b		
Clozapine	Schizophrenia, Suicidal	Tardive dyskinesia, agranulocytosis (older women), breast cancer, weight gain	Acute dystonia, abnormal ejaculation	5-HT1a	Antagonist	(49–51, 53)
				5-HT1b		
				5-HT1d		
				5-HT1e		
				5-HT2a		
				5-HT2c		
				5-HT6		
				5-HT7		
Fanaptum	Schizophrenia	Breast discharge, abnormal menstrual cycle, fertility decrease	Ejaculation failure	5-HT1a	Antagonist	(50, 52)
				5-H2a		
				5-HT6		
Flupentixol	Schizophrenia, Antidepressant	Amenorrhea, decreased fertility, hyperprolactinemia, galactorrhoea, decrease libido	Erectile dysfunction, ejaculation failure	5-HT2a	Antagonist	(50)
				5-HT2C		
Iloperidone	Schizophrenia	Galactorrhoea, menstruation irregular, menorrhagia, metrorrhagia, postmenopausal haemorrhage	Gynecomastia, erectile dysfunction, prostatitis	5-HT1a	Antagonist	(50, 56)
				5-HT2a		
				5-HT6		
				5-HT7		
Lumateperone Tosylate	Schizophrenia, Bipolar, Depression	Tardive dyskinesia (older women)	Acute dystonia	5-HT2a	Antagonist	(49, 50)
				5-HT2c		
Lurasidone	Bipolar 1, Schizophrenia	Amenorrhea, decreased lactation	Erectile dysfunction	5-HT1a	Antagonist	(50, 53, 57)
				5-HT1c		
				5-HT2a		
				5-HT7		
Olanzapine	Bipolar, Schizophrenia	Menstrual disorder, vaginal inflammation, vaginal discharge, menorrhagia, metrorrhagia, premenstrual syndrome, female lactation, uterine fibroids enlarged, vaginal haemorrhage, amblyopia, vaginitis	Erectile dysfunction	5-HT2a	Antagonist	(50)
				5-HT2c		
				5-HT6		
Paliperidone	Schizophrenia	Amenorrhea, decreased lactation, pregnancy risk increase	Erectile dysfunction	5-HT1a	Antagonist	(50, 51)
				5-HT1d		
				5-HT2a		
				5-HT2c		
				5-HT1a		
Quetiapine	Bipolar	Vaginal inflammation, female lactation, vaginal infection increase, vulvovaginitis, vaginal moniliasis, vaginitis, metrorrhagia, cystitis, amenorrhea, lactation, leukorrhea, vaginal haemorrhage, vulvovaginitis orchitis	Erectile dysfunction, abnormal ejaculation, gynecomastia	5-HT1a	Antagonist, partial agonist	(50, 53)
				5-HT2a	Antagonist	
				5-HT6		
Risperdal Consta	Schizophrenia, Bipolar Disorder	Tardive dyskinesia (elder women), thrombotic thrombocytopenic purpura, pituitary gland adenomas, impaired fertility, menstrual disorder, breast cancer, disorder and pain and vaginal inflammation, dryness and haemorrhage, unintended pregnancy, menstruation delayed, menstrual disorder, amenorrhea	Acute dystonia, erectile dysfunction, gynecomastia	5-HT1a	Antagonist	(49–51)
				5-HT1c		
				5-HT1d		
				5-HT2a		
				5-HT7		
Ziprasidone	Bipolar, Schizophrenia	Menorrhagia, metrorrhagia, female lactation, uterine and vaginal haemorrhage, sexual dysfunction, uterine haemorrhage, menorrhagia, female lactation, amenorrhea, metrorrhagia	Erectile dysfunction, abnormal ejaculation, male sexual dysfunction	5-HT1a	Agonist	(50, 51)
				5-HT1b		
				5-HT1d		
				5-HT1e		
				5-HT2a		
				5-HT2c		
				5-HT6		
				5-HT5a		
				5-HT7		
Citalopram	Anti-depressant	Breast pain, enlargement and discharge, endocrine disorder, amenorrhea, galactorrhoea, menstrual disorder, vaginal discharge, infection increase, dryness, inflammation, haemorrhage, pain and discharge, unintended pregnancy	Testicular disorder, testicular pain, ejaculation disorder	5-HT2a	Antagonist	(51, 52)
Imipramine Pamoate	Anti- Depressant	Breast enlargement, galactorrhoea	Gynecomastia, ineffectiveness, testicular swelling	5-HT1a	Agonist	(49–51)
				5-HT2a	Antagonist	
				5-HT2c		
				5-HT7		
				5-HT6	Partial agonist	
Nefazodone Hydrochloride	Anti-Depressant	Vaginitis, breast pain, uterine haemorrhage, uterine fibroids enlarged, menorrhagia	Galactorrhoea, erectile dysfunction, gynecomastia	5-HT1a	Antagonist	(49–51)
				5-HT2a		
				5-HT2c		
Clomipramine	OCD, MDD	Menstrual disorder, vaginal and uterine inflammation, ovarian cysts, uterine and vaginal haemorrhage, endometrial hyperplasia, uterine inflammation, amenorrhea, breast pain, breast enlargement, leukorrhea, vaginitis, dysmenorrhea	Erectile dysfunction, prostatic disorder, testicular swelling, impotence	5-HT2a	Antagonist	(52, 54, 58)
				5-HT2b		
				5-HT2c		
Escitalopram	MDD, GAD, OCD	Impair fertility, interference with fallopian tube motility, breast disorder, pain, enlargement and cancer risk, menstrual disorder, ovarian cyst, premenstrual disorder, vaginitis atrophic, vaginal discharge, infection increase, dryness, pain and haemorrhage	Ejaculation disorder, testicular pain, gynecomastia	5-HT1a	Antagonist	(50–52)
				5-HT2a		
				5-HT2c		
Paroxetine	MDD, Panic disorder, OCD, social phobia, GAD, PMDD, vasomotor, menopausal symptoms	Abdominal pain, attention disturbance, suicidal ideation, bone fractures, agitation, impaired fertility, interference with fallopian tube motility, menstrual disorder, breast pain, ovarian cyst, vaginal infection increase and inflammation, uterine spasm, uterine sertraline enlarged, endometrial disorder	Erectile dysfunction, testicular pain, breast atrophy, endometrial disorder, gynecomastia	5-HT2a	Agonist	(49–51)
				5-HT2b		
Nortriptyline	MDD	Galactorrhoea, breast enlargement	Erectile dysfunction	5-HT1a	Antagonist	(50)
				5-HT1c		
				5-HT2a		
				5-HT2b		
				5-HT2c		
Desipramine	MDD	Menstrual disorder, galactorrhoea, breast enlargement	Gynecomastia, erectile dysfunction	5-HT1a	Partial antagonist, agonist	(49)
				5-HT2a	Antagonist	
				5-HT2c	Partial antagonist, agonist	
Trazodone Hydrochloride	MDD	Lactation, clitorism	Priapism	5-HT1a	Antagonist	(49, 50)
				5-HT1c		
				5-HT2a	Antagonist, partial agonist	
				5-HT2b		
				5-HT2c		
Trimipramine Maleate	MDD	Galactorrhoea, breast enlargement	Gynecomastia, erectile dysfunction	5-HT1a	Antagonist	(49)
				5-HT2c		
				5-HT1d	Partial agonist, antagonist	
				5-HT2a	Agonist	
Vortioxetine	MDD	Sexual dysfunction	None reported	5-HT1a	Agonist	(50)
				5-HT1b	Partial agonist	
				5-HT7	Antagonist	
Almotriptan Malate	Migraine	Dysmenorrhea	None reported	5-HT1b	Agonist	(50, 51)
				5-HT1d		
Dihydroergotamine Mesylate	Migraine	Reproductive malfunction, pelvic inflammation, vaginitis, vaginal inflammation	None reported	5-HT1a	Agonist	(49–51)
				5-HT1d		
				5-HT2a		
				5-HT2c		
Eletriptan Hydrobromide	Migraine	Vaginal inflammation, menstrual disorder, menorrhagia	Erectile dysfunction	5-HT1a	Agonist	(50, 51)
				5-HT1b		
				5-HT1d		
				5-HT1f		
Frovatriptan	Migraine	None reported	None reported	5-HT1b	Agonist	(50)
				5-HT1d		
Methysergide Maleate	Migraine	Vasoconstrictive phenomena, hair loss, ineffectiveness	Mild temporal lobe disturbance	5-HT1a	Agonist	(49–51, 59)
				5-HT2a	Antagonist	
				5-HT2b		
				5-HT2c		
				5-HT7		
Naratriptan	Migraine	Vaginal inflammation, fallopian tube inflammation, endometrium disorder	None reported	5-HT1a	Agonist	(50)
				5-HT1b		
				5-HT1d		
				5-HT1f		
Sumatriptan	Migraine	Endometriosis, breast discharge, tenderness, lactation, cysts, lumps, abnormal menstrual cycle, breast cancer, dysmenorrhea, intermenstrual bleeding, abortion, menstruation symptoms, abnormal menstrual cycle, inflammation of fallopian tubes	None reported	5-HT1a	Agonist	(49–51)
				5-HT1b		
				5-HT1d		
				5-HT1f		
				5-HT5a		
				5-HT7		
Zolmitriptan	Migraine	Vaginal inflammation, uterine disorder, uterine fibroids enlarged	None reported	5-HT1a	Agonist	(50, 51)
				5-HT1b		
				5-HT1d		
				5-HT1e		
				5-HT1f		
				5-HT2a		
				5-HT2b		
				5-HT7		

Benign breast tumour is another common side effect of 5-HT GPCR-targeted drugs predominantly seen in women since prolactin promotes breast cell division (62) and prolonged hyperprolactinemia increases the risks of breast cancer. In cancer cells, constitutive prolactin activity promotes cell proliferation, cell motility, angiogenesis, and metastasis (63). Risperidone, sumatriptan, zolmitriptan and clozapine have both been shown to increase the risks of breast cancers (Supplementary Table S2). A cohort study showed breast cancer patients administered SSRIs have a 27% increase in mortality, showing that some antipsychotics contribute to breast cancer progression (64). Microarray analyses using a cancer cell line and immunohistochemistry using breast tumour tissues extracted from patients showed elevated accumulation of serotonin biosynthesis enzyme genes and proteins, respectively. In addition, *HTR7*, *HTR1D* and *HTR2B* genes encoding 5-HT GPCRs were also found upregulated in these samples (65). Serotonin is generally thought to promote the disease progression of breast cancer. The shift in expression of 5-HT GPCRs is believed to promote malignant pathways, such as cell proliferation and inhibition of cell cycle arrest. The above mentioned drugs identified to increase the risks of breast cancer (Supplementary Table S2) may be targeting the 5-HT7 receptor known to drive p38 mitogen-activated protein kinase (p38 MAPK) pathways that increases the chances of transforming breast cells to cancer cells (66).

Menstrual irregularities and infertility in women may also result from taking 5-HT GPCR-targeted drugs. A study using female mice receiving continuous infusions of prolactin to induce hyperprolactinemia showed prolonged elevated prolactin caused abrogation of the oestrous cycle and a decrease in FSH, luteinizing hormone (LH) and ovum lutea, resulting in anovulation (67). Without LH regulating the menstrual cycle and FSH causing female egg development, ovulation and menstruation do not take place, leading to infertility. As shown in Table 2 and Supplementary Table S2, side effects such as hyperprolactinemia and menstrual irregularities and/or infertility have been reported for drugs such as ziprasidone, olanzapine and risperidone. Taken together with the experimental results in mice, these drugs may cause menstrual irregularities and infertility as a result of hyperprolactinemia in women.

The effects of paroxetine and escitalopram oxalate have been tested on isolated isthmus and ampulla of 20 postmenopausal women who underwent hysterectomy (68). The tension of the isolated preparations was recorded by an isometric transducer and the effects of both drugs were recorded over time. The results showed that both paroxetine and escitalopram oxalate cause spontaneous contractions in the fallopian tube that may interfere with their natural function (68). A prolonged and uncontrolled fallopian tube spasm is associated with a decrease in fertility because it prevents sperm from being retained in the oviduct. Thus, the sperm cannot successfully fertilise the ovum (69). Although this is just one report, the above observation may explain the female reproductive impairment reported in women from taking paroxetine and escitalopram oxalate (Table 2).

A systematic literature review demonstrated that SSRIs altering sperm cell morphology cause a reduction in the number of viable sperm and, as a result, decrease male fertility (65). Hyperprolactinemia in male mice has been shown to inhibit the release of testosterone, which resulted in erectile dysfunction. Drugs, such as aripiprazole, flupentixol, risperidone and olanzapine have been reported to cause both hyperprolactinemia and erectile dysfunction (Table 2 and Supplementary Table S2).

### Interpretation

Across the board, the estimated number of adverse side effect cases reported in women is 1.5 to 1.7-fold greater than in men (65), which is consistent with the data published by Martin et al. (1998) (5). Thus, the ratio of women experiencing more adverse effects from medications has remained consistent over twenty years. In this review, we have investigated side effect reports, packet notes, and experimental results using laboratory animals and humans concerning drugs targeting serotonin 5-HT GPCRs and critically analysed the mechanism of the side effects in women and men (70).

Serotonin stored in the platelets in the cardiovascular system remains in the PNS, while serotonin in the CNS remains in the brain in the normal healthy body due to the blood-brain barrier (2). Here, we propose that those neurological drugs targeting 5-HT GPCRs in the CNS may interact with 5-HT GPCRs in the PNS and cause side effects within these areas. Expression patterns of 5-HT GPCRs in the PNS are localised in sexual organs and are, therefore, different between women and men. Due to the essential role of 5-HT GPCRs in sexual organs in humans, any disturbance of serotonin concentration in the PNS impacts the normal function of the respective sexual organs.

In both women and men, oestrogen regulates the expression of 5-HT GPCRs and serotonin biosynthesis (Figure 1). The promoters of the *TPH1* and *TPH2* genes have an ER binding element, and therefore oestrogen, and not androgen or testosterone, triggers serotonin biosynthesis (25, 26). When the oestrogen level is high, ER binding to the promoter of *HTR2A* results in upregulation of the 5-HT2a receptor. When oestrogen is elevated, ERß binds to the *HTR1A* promoter to inhibit transcription, thereby reducing the number of 5-HT1a receptors, whereas a decrease in oestrogen allows ERα binding to the *HTR1A* promoter, resulting in an increase of 5-HT1a receptors. A reduction of oestrogen results in a reduction of 5-HT2a receptors (28, 30). Compared to women, men produce lower levels of oestrogen and their oestrogen levels fluctuate less due to the absence of a menstrual cycle. Thus, it is assumed that oestrogen influences 5-HT GPCRs to a greater extent in women than in men. Studies have shown that serotonin biosynthesis and signalling pathways are affected preferentially by oestrogen, while there is very little evidence for male hormones such as androgen and testosterone having the same interaction. As a result, excitatory 5-HT GPCRs, such as 5-HT2 receptor types, can be hyper-activated when the oestrogen concentration is high (27, 32).

Results suggest neurological drugs interfere with the regulation of 5-HT GPCRs by oestrogen. A reduction and thus a potentially lower than anticipated level of serotonin concentration in the cardiovascular system leads to vasodilation of the extracerebral carotid arteries. This would increase the volume of blood flow in these arteries and increase pressure on the surrounding tissues in the centre of the brain causing pain as a result. The 5-HT1a receptor is known to induce vasoconstriction in the presence of the expected concentration of serotonin. There is a trough in serotonin concentration coinciding with the decrease in oestrogen during the menstrual cycle (Figure 2). This may explain why there are more women suffering from migraines and headaches that coincide with the time when oestrogen is at its lowest, i.e., prior to and during menstruation. Drugs treating bipolar disorder and schizophrenia are intended to act as an antagonist, targeting 5-HT2 receptors in the CNS. However, they may also bind to 5-HT1 receptors and could prevent vasoconstriction in the extracerebral carotid arteries if they remain in the PNS. Such antagonists, if given to women during pre-menstruation when oestrogen levels are at their lowest and 5-HT1a receptor expression is at its highest (Figure 2) during pre-menstruation, could cause severe and potentially debilitating headaches. This is because an inhibition of 5-HT1 receptors by 5-HT GPCR antagonists would result in vasodilation. One suggestion to be considered is to reduce the dosage or end administration of 5-HT GPCR antagonists to women who are bipolar or schizophrenic during phases of the menstrual cycle when oestrogen is at its lowest.

Although drugs for the treatment of dravets syndrome and other conditions, such as bipolar and schizophrenia, target 5-HT GPCR, those used to treat the former do not have a large number of reported side effect cases (Supplementary Table S2). Dravets syndrome, in most cases, is diagnosed and treated in infants, whereas schizophrenia and bipolar are diagnosed and given to patients post puberty (15, 17). Thus, drugs for dravets syndrome are less likely to interfere with the oestrogen-mediated serotonin system to the same extent as in sexually matured and adult patients treated with other 5-HT GPCR drugs. This could explain why erectile dysfunction, menstrual irregularities, lactation or breast pain are not commonly reported side effects of drugs treating dravet syndrome patients, while it is for those taking antipsychotics. The impact of drugs targeting 5-HT GPCRs on sexual hormones could be a major cause of reported side effects.

The selective serotonin reuptake inhibitor, fluvoxamine, known to increase serotonin concentrations in the brain, results in a decrease in *β-casein* expression in breast cells (MCF-12A) (71). These results suggest that agonists to excitatory 5-HT GPCRs, which artificially promote the downstream G-protein signalling, are likely to be suppressing production of the milk protein, β-casein and thereby preventing milk production. Women who breastfeed and take antipsychotics, such as aripiprazole, could experience a delayed activation of milk secretion. We noticed that not all packet notes of antipsychotics have this warning. However, as all antipsychotics could affect milk secretion, clinicians could inform those breastfeeding and pregnant patients of the potential for delay and reduction in milk production.

In a study conducted by the Institute of Psychiatry, sexual dysfunction experienced by patients taking antipsychotics is more commonly seen in women than in men (15.2: 3.7 ratio between women and men, respectively *n* = 103) (71). The two major consequences affecting women and men taking 5-HT targeted drugs are the changing number of 5-HT GPCRs affected by a given oestrogen concentration and hyperprolactinemia. Infertility caused by 5-HT targeted drugs may be experienced more in women than in men due to the changing levels of oestrogen during the menstrual cycle. The risk of infertility may be heightened in women when oestrogen concentrations are low and thus excitatory 5-HT GPCRs decrease, causing reduced muscle contractions in the fallopian tube, uterus, and cervix, essential for fertility. During this time, agonists to inhibitory 5-HT GPCRs or antagonists to excitatory 5-HT GPCRs may further inhibit serotonin's influence on fertility. Hyperprolactinemia in women will affect ovule production and ovulation and manifest in men as a decrease in sperm production. Patients taking 5-HT GPCR-targeting drugs should be informed of potential infertility. Hyperprolactinemia and elevated 5-HT1d, 5-HT2b and 5-HT7 receptor activity are both linked to a higher risk of developing breast cancer due to their positive role in cell proliferation (63, 66). One of the most common side effects of 5-HT GPCR-targeting drugs is hyperprolactinemia (Supplementary Table S2). Due to the chances of infertility and developing cancer, blood prolactin levels could be monitored.

Women are subject to cycles of dynamic change in concentrations of serotonin compared to men through oestrogen hormonal activation of serotonin biosynthesis (Figure 1). Thus, when oestrogen is at its highest during ovulation until the mid-luteal phase, the endogenous serotonin concentration is expected to be relatively high. In order to reduce the side effect, agonists to excitatory 5-HT receptors or antagonists to inhibitory 5-HT receptors could be administered during the menstrual period when the oestrogen concentration is at its lowest.

Serotonin metabolism differs between men and women. Animal testing of most drugs has been conducted preferentially on male animals due to their consistency and the relatively low deviation in the results. Given that the 5-HT GPCR-targeting drugs are likely metabolised slower in women than in men, the absorption, distribution, metabolism and excretion (ADME) of each drug needs to be tested using female animals. Generally, women have a lower biological concentration of serotonin. Therefore, we should consider administering lower doses of 5-HT GPCR-targeting drugs to women.

In conclusion, more female mouse models can be used during phase II of drug development when testing for those targeting 5-HT GPCRs. Secondly, blood prolactin concentration can be monitored following administration of 5-HT GPCR-targeting drugs in both women and men. Lastly, we could take into consideration the timing of 5-HT GPCR-targeting drug administration in relation to cycling oestrogen due to the menstrual cycle in women.
