# Supplementary material for: Recent advances in understanding adverse effects associated with drugs targeting the serotonin receptor, 5-HT GPCR
Source: Front Glob Womens Health. 2022 Dec 8;3:1012463. doi: 10.3389/fgwh.2022.1012463 (PMC9812521; doi:10.3389/fgwh.2022.1012463)
Supplement: Supplementary file 1 [file Table1.docx]

**Supplementary Table 1**. Drug names and PDF packet insert from either FDA or EMA.

| **Drug name** | **PDF URL** |
| --- | --- |
| Almotriptan Malate | https://www.accessdata.fda.gov/drugsatfda_docs/label/2009/021001s010s011lbl.pdf |
| Amisulpride | https://www.accessdata.fda.gov/drugsatfda_docs/label/2020/209510s000lbl.pdf |
| Aripiprazole | <https://www.ema.europa.eu/en/documents/product-information/aripiprazole-mylan-pharma-epar-product-information_en.pdf> |
| Cariprazine | <https://www.accessdata.fda.gov/drugsatfda_docs/label/2015/204370lbl.pdf> |
| Cisapride | <https://www.accessdata.fda.gov/drugsatfda_docs/nda/2000/20767-S011_Propulsid.pdf> |
| Citalopram | [https://www.accessdata.fda.gov/drugsatfda_docs/label/2009/020822s037,021046s015lbl.pdf](https://www.accessdata.fda.gov/drugsatfda_docs/label/2009/020822s037,021046s015lbl.pdf ) |
| Clomipramine | https://www.accessdata.fda.gov/drugsatfda_docs/label/2007/019906s34lbl.pdf |
| Clozapine | <https://www.accessdata.fda.gov/drugsatfda_docs/label/2014/019758s073lbl.pdf> |
| Cyproheptadine Hydrochloride | [https://www.accessdata.fda.gov/drugsatfda_docs/label/2009/087056s045lbl.pdf](https://www.accessdata.fda.gov/drugsatfda_docs/label/2009/087056s045lbl.pdf ) |
| Desipramine | https://www.accessdata.fda.gov/drugsatfda_docs/label/2014/014399s069lbl.pdf |
| Dihydroergotamine Mesylate | <https://www.accessdata.fda.gov/drugsatfda_docs/label/2019/020148Orig1s025lbl.pdf> |
| Eletriptan Hydrobromide | https://www.accessdata.fda.gov/drugsatfda_docs/label/2013/021016s021s023s024s027lbl.pdf |
| Escitalopram | https://www.medicines.org.uk/emc/files/pil.7059.pdf |
| Fanaptum | https://www.ema.europa.eu/en/documents/assessment-report/fanaptum-epar-refusal-public-assessment-report_en.pdf |
| Fenfluramine Hydrochloride | https://www.ema.europa.eu/en/documents/product-information/fintepla-epar-product-information_en.pdf |
| Flupentixol | https://www.accessdata.fda.gov/drugsatfda_docs/nda/2016/125521orig1s000medr.pdf |
| Frovatriptan | https://www.accessdata.fda.gov/drugsatfda_docs/label/2009/021006s006s009s010lbl.pdf |
| Iloperidone | https://www.accessdata.fda.gov/drugsatfda_docs/label/2016/022192s017lbl.pdf |
| Imipramine Pamoate | <https://www.accessdata.fda.gov/drugsatfda_docs/label/2014/017090s078lbl.pdf> |
| Lumateperone Tosylate | <https://www.accessdata.fda.gov/drugsatfda_docs/label/2019/209500s000lbl.pdf> |
| Lurasidone | https://www.accessdata.fda.gov/drugsatfda_docs/label/2013/200603lbls10s11.pdf |
| Methysergide | https://www.ema.europa.eu/en/documents/referral/methysergide-article-31-referral-assessment-report_en.pdf |
| Naratriptan | https://www.accessdata.fda.gov/drugsatfda_docs/label/1998/20763lbl.pdf |
| Nefazodone Hydrochloride | <https://www.accessdata.fda.gov/drugsatfda_docs/label/2014/076037s011lbl.pdf> |
| Nortriptyline | https://www.accessdata.fda.gov/drugsatfda_docs/label/2007/018013s58lbl.pdf |
| Olanzapine | https://www.accessdata.fda.gov/drugsatfda_docs/label/2014/020592s062021086s040021253s048lbl.pdf |
| Paliperidone | https://www.accessdata.fda.gov/drugsatfda_docs/label/2010/021999s018lbl.pdf |
| Paroxetine | https://www.accessdata.fda.gov/drugsatfda_docs/label/2017/204516s004lbl.pdf |
| Quetiapine | https://www.accessdata.fda.gov/drugsatfda_docs/label/2004/20639se1-017,016_seroquel_lbl.pdf |
| Risperdal Constra | https://www.accessdata.fda.gov/drugsatfda_docs/label/2010/021346_s31_s35_s38_s39lbl.pdf |
| Sumatriptan | <https://www.accessdata.fda.gov/drugsatfda_docs/label/2012/020132s024s026lbl.pdf> |
| Trazodone Hydrochloride | <https://www.accessdata.fda.gov/drugsatfda_docs/label/2017/018207s032lbl.pdf> |
| Trimipramine Maleate | https://www.accessdata.fda.gov/drugsatfda_docs/label/2012/016792s034lbl.pdf |
| Vortioxetine | https://www.accessdata.fda.gov/drugsatfda_docs/label/2018/204447s017lbl.pdf |
| Ziprasidone | https://www.accessdata.fda.gov/drugsatfda_docs/label/2014/020825s053,020919s040,s021483s013lbl.pdf |
| Zolmitriptan | https://www.accessdata.fda.gov/drugsatfda_docs/label/2012/020768s019s021,021231s010s011lbl.pdf |
