# Supplementary material for: Recent advances in understanding adverse effects associated with drugs targeting the serotonin receptor, 5-HT GPCR
Source: Front Glob Womens Health. 2022 Dec 8;3:1012463. doi: 10.3389/fgwh.2022.1012463 (PMC9812521; doi:10.3389/fgwh.2022.1012463)
Supplement: Supplementary file 2 [file Table2.docx]

**Supplementary Table 2 represents drugs used to treat psychological, neurological conditions that target serotonin receptors, their side effects in both sexes, women more than men, in men more than women.** Data in Table 2 showing frequent, infrequent and rare side effects is obtained from packet inserts listed in Supplementary Table 1 from FDA, EMA drug approval bodies, SIDER and DrugBank. Abbreviations: MDD: Multiple Personality Disorder, OCD: Obsessive compulsive Disorder, GAD: Generalized anxiety disorder, PMDD: Premenstrual dysphoric disorder, SAD: social anxiety disorder, PD: panic disorder and 5-HT: serotonin, NMS: neuroleptic malignant syndrome ADH: antidiuretic hormone UTI: urinary tract infection, Orange coloured cells represent excitatory receptors, blue represents inhibitory receptors.

| **Generic   Drug Name** | **Treatment** | **Side effects in both sexes** | **Side effect seen more in women**  **than in men** | **Side effect seen more in men than in women** | **5-HT receptor Target** | **Antagonist**  **/Agonist** | **References** |
| --- | --- | --- | --- | --- | --- | --- | --- |
| **Cisapride** | Anti-heart burn | sinusitis, coughing, body pain, constipation, diarrhea, weight loss, lack of appetite, confusion, abdominal pain, nausea, flatulence dyspepsia, rhinitis, viral infection, upper respiratory tract infection, fever, UTI, micturition frequency, insomnia, anxiety, nervousness, rash, purinitis, arthralgia, abnormal vision, dizziness, vomiting, pharyngitis, chest pain, back pain, depression, dehydration, myalgia, cardiac arrhythmias, ventricular tachycardia, ventricular fibrillation, torsades de pointes, hallucination, akathisia, Parkinson-like symptoms, dyskinetic dystonic reactions, breast enlargement, urinary incontinence, hyperprolactinemia, antinuclear antibody positive, anaemia, hemolytic anemia, methemoglobinemia, hyperglycemia, hypoglycemia with acidosis, severe photosensitivity reaction, suicide attempt, bronchospasm, urticaria, angioedema asthma | fatal heart disturbance, vaginitis, reproductive failure, galactorrhea | gynecomastia | 5-HT2a | Agonist | (61, 62) |
|  |  |  |  |  | 5-HT2c |  |  |
|  |  |  |  |  | 5-HT4 |  |  |
| **Cyproheptadine Hydrochloride** | Hypersensitivity Reactions symptoms | sedation, sleepiness (often transient), dizziness, disturbed coordination, confusion, restlessness, excitation, nervousness, tremor, irritability, insomnia, paresthesia’s, neuritis, convulsions, euphoria, hallucinations, hysteria, faintness, allergic manifestation of rash, edema, excessive perspiration, urticaria, photosensitivity, acute labyrinthitis, blurred vision, diplopia, vertigo, tinnitus, hypotension, palpitation, tachycardia, extrasystoles, anaphylactic shock, hemolytic anemia, leukopenia, agranulocytosis, thrombocytopenia, cholestasis, hepatic failure, hepatitis, hepatic function abnormality, dry mouth, epigastric distress, anorexia, nausea, vomiting, diarrhea, constipation, jaundice, urinary frequency, difficult urination, urinary retention, early menses, dryness of nose, throat, thickening of bronchial secretions, tightness of chest, wheezing, nasal stuffiness | none reported | none reported | 5-HT2a | Antagonist | (61-63) |
|  |  |  |  |  | 5-HT2b |  |  |
|  |  |  |  |  | 5-HT2c |  |  |
|  |  |  |  |  | 5-HT7 |  |  |
| **Fenfluramine Hydrochloride** | Dravet Syndrome | increase in blood pressure, serotonin syndrome, withdrawal of antiepileptic drugs, suicidal behavior, ideation, somnolence, sedation, lethargy, glaucoma, decreased appetite, diarrhea, constipation, abnormal echocardiogram, fatigue, malaise, asthenia, ataxia, balance disorder, gait disturbance, drooling, salivary hypersecretion, pyrexia, vomiting, decreased weight, fall, status epilepticus, bronchitis, upper respiratory tract infection, tremor, blood glucose decreased | none reported | none reported | 5-HT1a | Agonist | (62, 64) |
|  |  |  |  |  | 5-HT1d |  |  |
|  |  |  |  |  | 5-HT2a |  |  |
|  |  |  |  |  | 5-HT2b |  |  |
|  |  |  |  |  | 5-HT2c |  |  |
| **Amisulpride** | Schizophrenia, PONV | breast growth, lactation, endocrine disorder, anxiety, insomnia, constipation, diarrhea, tremor, salivary hypersecretion, weight change, increase infection risk, sweating, hyperprolactinemia, dizziness, somnolence, headache, nausea, vomiting, dry mouth, paresthesia, chest pain, tightness, pressure in the chest, or throat, neck, jaw, cerebral haemorrhage, subarachnoid haemorrhage, stroke, peripheral vascular ischeamia, colonic ischeamia, serotonin syndrome, hypersensitivity to sulfonamides, impaired hepatic or renal function, long-term ophthalmologic effects, corneal opacities, anaphylactic shock, confusional state, restlessness, hemiplegia, seizures blepharospasm, vertigo, acute myocardial infarction, coronary artery vasospasm, angina pectoris, tachycardia, abdominal discomfort, abdominal pain, colitis, hypoesthesia-oral, swollen tongue, angioedema, cold sweat, erythema, hyperhidrosis, arthralgia, myalgia, pain in extremity, breast pain, malaise, peripheral coldness | vaginal inflammation, menstrual disorder, pregnancy risk increase, infertility | ejaculatory dysfunction | 5-HT2a | Antagonist | (62, 64-66) |
|  |  |  |  |  | 5-HT2b |  |  |
|  |  |  |  |  | 5-HT7 |  |  |
| **Aripiprazole Mylan** | Schizophrenia, Bipolar I | akathisia, hyperprolactinemia, libido increase or decrease increased blood prolactin concentrations, chills, hypokalemia, abdominal distension, lymphatic system disorders, agranulocytosis, bradycardia, torsades de pointes, ventricular tachycardia, prolonged qt by electrocardiogram, neuroleptic malignant syndrome, angioedema, urticaria, increased hepatic enzymes, agitation, anxiety, dystonia, extrapyramidal disorder, seizure, confusional state, insomnia, hypotension, suicidality, cardiovascular disorders, tardive dyskinesia, NMS, seizures, increased mortality, mortalitvasculary, stroke, transient ischaemic attack, hyperglycemia, diabetes mellitus, hypersensitivity, weight gain, dysphagia, impulse control disorders, somnolence, motor, sensory instability, insomnia, restlessness, vision blurred | decreased libido, tardive dyskinesia (older women), weight gain, | erectile dysfunction, dystonia | 5-HT1a | Partial agonist | (61, 62, 64, 67) |
|  |  |  |  |  | 5-HT1b | Antagonist |  |
|  |  |  |  |  | 5-HT1d |  |  |
|  |  |  |  |  | 5-HT2a |  |  |
|  |  |  |  |  | 5-HT2c | Antagonist, partial agonist |  |
|  |  |  |  |  | 5-HT7 |  |  |
| **Cariprazine Hydrochloride** | Schizophrenia, Bipolar | extrapyramidal symptoms, akathisia, dyspepsia, vomiting, somnolence, restlessness, tardive dyskinesia, leukopenia, neutropenia, agranulocytosis, orthostatic hypotension, syncope, seizures, dysregulation, dysphagia, NMS, including stroke, increased mortality in elderly patients with dementia-related psychosis, hyperglycemia, diabetes mellitus, dyslipidemia, weight gain, leukopenia, neutropenia, agranulocytosis, orthostatic hypotension, syncope, cognitive, motor impairment, body temperature dysregulation, dysphagia, diarrhea, abdominal pain, nausea, constipation, flatulence, rhinitis, sinusitis, coughing, viral infection, upper respiratory tract infection, body pain, fever, UTI, micturition frequency, insomnia, anxiety, nervousness, skin, appendages, rash, pruritus, arthralgia, abnormal vision | tardive dyskinesia (older women), decrease fertility, amenorrhea, vaginitis | ejaculation dysfunction | 5-HT1a | Partial agonist | (18, 61, 62) |
|  |  |  |  |  | 5-HT2a | Antagonist |  |
|  |  |  |  |  | 5-HT2b |  |  |
| **Clozapine** | Schizophrenia, Suicidal | vertigo, hyperprolactinemia, agranulocytosis, myocarditis, drowsiness/sedation, dizziness, headache, tremor, syncope disturbed sleep/nightmares restlessness, hypokinesia/akinesia, agitation seizures (convulsions), rigidity, akathisia, confusion, fatigue, insomnia, hyperkinesia, instability, lethargy, ataxia, slurred speech, depression, epileptiform movements/myoclonic jerks, anxiety, tachycardia hypotension hypertension chest pain/angina ECG change/cardiac abnormality, constipation, nausea, abdominal, discomfort/heartburn, vomiting, diarrhea, liver test abnormality, anorexia, urinary abnormalities, incontinence, abnormal ejaculation, urgency/frequency, urinary retention, autonomic nervous system salivation, sweating, dry mouth, visual disturbances, rash, muscle weakness, pain (back, neck, legs), muscle spasm, muscle pain, ache, throat discomfort, dyspnea, shortness of breath, nasal congestion, leukopenia/decreased WBC/neutropenia, agranulocytosis, eosinophilia, fever, weight gain, tongue numb/sore | tardive dyskinesia, agranulocytosis (older women), breast cancer, weight gain | acute dystonia, abnormal ejaculation | 5-HT1a | Antagonist | (61-63, 65) |
|  |  |  |  |  | 5-HT1b |  |  |
|  |  |  |  |  | 5-HT1d |  |  |
|  |  |  |  |  | 5-HT1e |  |  |
|  |  |  |  |  | 5-HT2a |  |  |
|  |  |  |  |  | 5-HT2c |  |  |
|  |  |  |  |  | 5-HT6 |  |  |
|  |  |  |  |  | 5-HT7 |  |  |
| **Fanaptum** | Schizophrenia | tachycardia, diarrhea, arrhythmia, decrease blood pressure, nausea, dizziness, somnolence, renal failure, cardiac failure, cardio-respiratory failure, diabetes mellitus, pneumonia, pylorus occlusion, septicemia, suicide, cardiac arrest, volvulus, akathisia, extrapyramidal symptoms, seizures, eye disorder, weight increase, decreased appetite, back pain, muscle rigidity, headache, tremor, dyskinesia, dystonia, extrapyramidal disorder, salivation, bradykinesia, anxiety agitation, insomnia, delusion, depression hallucinations, restlessness, hostility | breast discharge, abnormal menstrual cycle, fertility decrease | ejaculation failure | 5-HT1a | Antagonist | (62, 64) |
|  |  |  |  |  | 5-HT2a |  |  |
|  |  |  |  |  | 5-HT6 |  |  |
| **Flupentixol** | Schizophrenia, antidepressant | hyperprolactinemia, galactorrhea, libido decreased, insomnia, urinary retention, hyperhidrosis, pruritus, akathisia, hyperkinesia, hypokinesia, dyspnea, cellulitis, appendicitis, depression, chronic obstructive pulmonary disease, Crohn’s disease, suicide attempt erysipelas, prostatitis, bipolar disorder, fall, abscess oral, anxiety, arthritis, biliary colic, cholecystitis, dehydration, urosepsis, urinary tract infection, type 2 diabetes mellitus, tonsillitis, thoracic vertebral fracture, retinal detachment, pyelonephritis acute, pustular psoriasis, pancreatitis acute, invasive ductal breast carcinoma, epilepsy, colitis ischaemic, cerebral artery embolism, bronchopneumonia, atrioventricular block first degree, arterial fibrillation, angioedema, inguinal hemia, acute myocardial infraction, urticaria, subdural haematoma, skin lesion, renal impairment, peritonitis, oesophagitis, lumbar radiculopathy, hypersensitivity vasculitis, hepatic function abnormal, gastritis, duodenitis | amenorrhea, decreased fertility | erectile dysfunction, ejaculation failure | 5-HT2a | Antagonist | (62) |
|  |  |  |  |  | 5-HT2C |  |  |
| **Iloperidone** | Schizophrenia | tachycardia, diarrhea, weight change, tremor, lethargy, akathisia, back pain, fatigue, musculoskeletal stiffness, vision blurred nausea, dry mouth, diarrhea, abdominal discomfort nasopharyngitis, upper respiratory tract infection, dizziness, somnolence, extrapyramidal disorder, tremor, lethargy, nasal congestion, dyspnea, rash, orthostatic hypotension, hypotension akathisia, bradykinesia, dyskinesia, dystonia, parkinsonism, breast pain, anemia, iron deficiency, leukopenia, palpitations, arrhythmia, atrioventricular block first degree, cardiac failure (including congestive, acute), vertigo, tinnitus,  hypothyroidism , conjunctivitis (including allergic), dry eye, blepharitis, eyelid edema, eye swelling, lenticular opacities, cataract, hyperemia (including conjunctival),  gastritis, salivary hypersecretion, fecal incontinence, aphthous stomatitis, duodenal ulcer, hiatus hernia, hyperchlorhydria, lip ulceration, reflux esophagitis, edema (general, pitting, due to cardiac disease), difficulty in walking, thirst, hyperthermia, cholelithiasis weight decreased, hemoglobin decreased, neutrophil count increased, hematocrit decreased, increased appetite, dehydration, hypokalemia, fluid retention, myalgia, muscle spasms, paresthesia, psychomotor hyperactivity, restlessness, amnesia, nystagmus, restless legs syndrome, restlessness, aggression, delusion, hostility, libido decreased, paranoia, anorgasmia, confessional state, mania, catatonia, mood swings, panic attack, obsessive-compulsive disorder, bulimia nervosa, delirium, polydipsia psychogenic, impulse-control disorder, major depression, urinary incontinence, dysuria, pollakiuria, enuresis, nephrolithiasis, urinary retention, renal failure acute, epistaxis, asthma, rhinorrhea, sinus congestion, nasal dryness, dry throat, sleep apnea syndrome, dyspnea exertional | galactorrhea, irregular menstruation, menorrhagia, metrorrhagia, postmenopausal heamorrhage, | gynecomastia erectile dysfunction, prostatitis | 5-HT1a | Antagonist | (62, 68) |
|  |  |  |  |  | 5-HT2a |  |  |
|  |  |  |  |  | 5-HT6 |  |  |
|  |  |  |  |  | 5-HT7 |  |  |
| **Lumateperone Tosylate** | Schizophrenia, Bipolar, Depression | somnolence/sedation, nausea, dry mouth, dizziness, creatine phosphokinase, fatigue, vomiting, hepatic transaminases, decreased appetite, increased mortality in elderly patients with dementia-related psychosis, stroke, transient ischaemic attack hyperpyrexia, muscle rigidity, delirium, autonomic instability, additional signs may include elevated, myoglobinuria (rhabdomyolysis), acute renal failure, NMS, tardive dyskinesia, hyperglycemia, diabetes mellitus, dyslipidemia, weight gain, leukopenia, neutropenia, agranulocytosis, orthostatic hypotension, syncope, falls, seizures, potential for cognitive, motor impairment, body temperature dysregulation, dysphagia | tardive dyskinesia (older women) | acute dystonia | 5-HT2a | Antagonist | (61, 62) |
|  |  |  |  |  | 5-HT2c |  |  |
| **Lurasidone** | Bipolar 1, schizophrenia | breast pain, breast disorder, breast enlargement, akathisia, insomnia, diarrhea, rash, UTI, infection increase risk, anemia, vertigo, hyperprolactinemia, NMS, hyperpyrexia, muscle rigidity, altered mental status, evidence of autonomic instability (irregular pulse or blood pressure, tachycardia, diaphoresis, cardiac dysrhythmia), creatinine phosphokinase, myoglobinuria (rhabdomyolysis), acute renal failure, tardive dyskinesia, hyperglycemia, diabetes mellitus, dyslipidemia, weight gain, leukopenia, neutropenia, agranulocytosis, orthostatic hypotension, syncope, seizures, potential for cognitive, motor impairment, body temperature dysregulation, suicide, activation of mania/hypomania, dysphagia | amenorrhea, decreased lactation | erectile dysfunction | 5-HT1a | Antagonist | (62, 65, 69) |
|  |  |  |  |  | 5-HT1c |  |  |
|  |  |  |  |  | 5-HT2a |  |  |
|  |  |  |  |  | 5-HT7 |  |  |
| **Olanzapine** | Bipolar, schizophrenia | hyperprolactinemia, breast pain, growth, libido increase postural hypotension, personality disorder, akathisia, fatigue, asthenia, somnolence, tremor, sedation, weight increased, increased appetite, headache, fatigue, dizziness, abdominal pain, pain in extremity, dry mouth, weight gain, back pain, constipation, speech disorder, increased salivation, amnesia, paresthesia, suicide, NMS, hyperpyrexia, muscle rigidity, altered mental status, evidence of autonomic instability (irregular pulse or blood pressure, tachycardia, diaphoresis, cardiac dysrhythmia), elevated creatinine phosphokinase, myoglobinuria (rhabdomyolysis), acute renal failure, pneumonia, systemic infection, hyperglycemia, diabetes mellitus, dyslipidemia, tardive dyskinesia, orthostatic hypotension, leukopenia, neutropenia, agranulocytosis, dysphagia, seizures, cognitive, motor impairment, body temperature regulation, ecchymosis, peripheral edema, increased appetite, joint pain, somnolence, insomnia, dizziness, urinary incontinence, urinary tract infection, speech disorder, amnesia, pharyngitis, dyspnea, amblyopia | menstrual disorder, vaginal inflammation, vaginal discharge, menorrhagia, metrorrhagia, premenstrual syndrome, female lactation, uterine fibroids enlarged, vaginal haemorrhage, amblyopia, vaginitis | erectile dysfunction | 5-HT2a | Antagonist | (62) |
|  |  |  |  |  | 5-HT2c |  |  |
|  |  |  |  |  | 5-HT6 |  |  |
| **Paliperidone** | Schizophrenia | hyperprolactinemia, breast disorder, breast cancer, endocrine disorder, sexual dysfunction, anorgasmia, pregnancy risk increase, decreased libido, cerebrovascular adverse reactions, including stroke, in elderly patients with dementia-related psychosis, NMS, hyperpyrexia, muscle rigidity, altered mental status, evidence of autonomic instability (irregular pulse or blood pressure, diaphoresis, cardiac dysrhythmia), tardive dyskinesia, hyperglycemia, diabetes mellitus, gastrointestinal obstruction, orthostatic hypotension, syncope, leukopenia, neutropenia, agranulocytosis, cognitive, motor impairment, seizures, dysphagia, suicide, thrombotic thrombocytopenic purpura (TTP), body temperature regulation, antiemetic effect, abdominal pain, dry mouth, salivation, fatigue, akathisia, dizziness, extrapyramidal symptoms, headache, somnolence, tachycardia, sinus arrhythmia, bundle branch block, atrioventricular block first, constipation, dyspepsia, asthenia, nasopharyngitis, rhinitis, upper respiratory tract infection, back pain, myalgia, akathisia, dysarthria, extrapyramidal symptoms, cough, bradycardia, anaphylactic reaction, urinary tract infection, electrocardiogram abnormal, arthralgia, edema, swollen tongue, nasal congestion, pneumonia aspiration, hypotension, convulsion, lethargy, syncope, nasal congestion, pneumonia aspiration | amenorrhea, decreased lactation, pregnancy risk increase | erectile dysfunction, priapism | 5-HT1a | antagonist | (62, 63) |
|  |  |  |  |  | 5-HT1d |  |  |
|  |  |  |  |  | 5-HT2a |  |  |
|  |  |  |  |  | 5-HT2c |  |  |
|  |  |  |  |  | 5-HT1a |  |  |
| **Quetiapine** | Bipolar | libido increase or decrease, breast disorder, growth malnutrition, constipation, insomnia, tachycardia, diarrhea, anorexia, increase infection risk, hormonal disorder, hypersomnia anxiety, UTI, back pain, hyperprolactinemia seizures, hypothyroidism, cholesterol, triglyceride elevations, transaminase elevations, motor impairment, priapism, irregular body temperature regulation, dysphagia, suicide, orthostatic hypotension, abnormal cognitive, motor performance, dehydration, carcinogenesis, mutagenesis, impaired infertility, headache, asthenia, abdominal pain, fever, postural hypotension, dry mouth, vomiting, dyspepsia, gastroenteritis, gamma glutamyl transpeptidase increased, weight gain, SGPT increased, SGOT increased, agitation, somnolence, dizziness, anxiety, pharyngitis, rhinitis, rash, amblyopia, aphasia, buccoglossal syndrome, choreoathetosis, delirium, emotional lability, euphoria, neuralgia, stuttering, subdural hematoma, abnormal dreams, thinking abnormal, tardive dyskinesia, vertigo, involuntary movements, confusion, amnesia, psychosis, hallucinations, hyperkinesia, urinary retention, incoordination, paranoid reaction, abnormal gait, myoclonus, delusions, manic reaction, apathy, ataxia, depersonalization, stupor, bruxism, catatonic reaction, hemiplegia, hypertonia, dysarthria, flu syndrome, neck pain, pelvic pain, suicide attempt, malaise, photosensitivity reaction, chills, face edema, moniliasis, abdomen enlarged, anorexia, increased salivation, increased appetite, gamma glutamyl transpeptidase increased, gingivitis, dysphagia, flatulence, gastroenteritis, gastritis, haemorrhoids, stomatitis, thirst, tooth caries, fecal incontinence, gastroesophageal reflux, gum haemorrhage, mouth ulceration, rectal haemorrhage, tongue edema, glossitis, haematemesis, intestinal obstruction, melena, pancreatitis, palpitation, vasodilatation, qt interval prolonged, migraine, bradycardia, cerebral ischeamia, irregular pulse, t wave abnormality, bundle branch block, cerebrovascular accident, deep thrombophlebitis, t wave inversion, angina pectoris, atrial fibrillation, av block first degree, congestive heart failure, ST elevated, thrombophlebitis, ST abnormality, increased QRS duration, pharyngitis, rhinitis, cough increased, dyspnea, pneumonia, epistaxis, asthma, hiccup, hyperventilation, peripheral edema, weight loss, alkaline phosphatase increased, hyperlipemia, alcohol intolerance, dehydration, hyperglycemia, creatinine increased, hypoglycemia, glycosuria, gout, hand edema, hypokalemia, water intoxication, sweating, pruritus, acne, eczema, contact dermatitis, maculopapular rash, seborrhea, skin ulcer, exfoliative dermatitis, psoriasis, skin discoloration, nocturia, polyuria, acute kidney failure, conjunctivitis, abnormal vision, dry eyes, tinnitus, taste perversion, blepharitis, eye pain, abnormality of accommodation, deafness, glaucoma, pathological fracture, myasthenia, twitching, arthralgia, arthritis, leg cramps, bone pain, leukopenia, leukocytosis, anemia, ecchymosis, eosinophilia, hypochromic anemia, lymphadenopathy, cyanosis, hemolysis, thrombocytopenia, hypothyroidism, diabetes mellitus, hyperthyroidism, dysmenorrhea, urinary incontinence, impotence, dysuria, urinary frequency | vaginal inflammation, female lactation, vaginal haemorrage, vaginal infection increase, vulvovaginitis, vaginal moniliasis, vaginitis, metrorrhagia, cystitis, amenorrhea, lactation, leukorrhea, vaginal haemorrhage, vulvovaginitis orchitis, | erectile dysfunction, abnormal ejaculation, gynecomastia | 5-HT1a | Antagonist, partial agonist | (62, 65) |
|  |  |  |  |  | 5-HT2a | Antagonist |  |
|  |  |  |  |  | 5-HT6 |  |  |
| **Risperdal Consta** | Schizophrenia, Bipolar Disorder | serotonin syndrome, hyperprolactinemia, headache, parkinsonism, dizziness, akathisia, fatigue, constipation, dyspepsia, sedation, weight increased, pain in extremity, dry mouth, cerebrovascular adverse events, including stroke, in elderly patients with dementia-related psychosis, NMS are hyperpyrexia, muscle rigidity, altered mental status, evidence of autonomic instability (irregular pulse or blood pressure, tachycardia, diaphoresis, cardiac dysrhythmia), tardive dyskinesia, hyperglycemia, diabetes mellitus, orthostatic hypotension, leukopenia, agranulocytosis, cognitive, motor impairment, seizures, dysphagia, priapism, thrombotic thrombocytopenic purpura (TTP), disruption of body temperature regulation, suicide, antiemetic effect, osteodystrophy, tumors, anemia, neutropenia, atrioventricular block first degree, palpitations, block left, bradycardia, bundle branch block right ear pain, vertigo, hyperprolactinemia, conjunctivitis, visual acuity reduced, diarrhea, vomiting, abdominal pain upper, abdominal pain, stomach discomfort, gastritis, injection site pain, chest discomfort, chest pain, influenza-like illness, sluggishness, malaise, induration, injection site induration, injection site swelling, injection site reaction, face edema, hypersensitivity, nasopharyngitis, influenza, bronchitis, urinary tract infection, rhinitis, respiratory tract infection, ear infection, pneumonia, lower respiratory tract infection, pharyngitis, sinusitis, viral infection, infection, localized infection, cystitis, gastroenteritis, subcutaneous abscess, fall, procedural pain, blood prolactin increased, alanine aminotransferase increased, electrocardiogram abnormal, gamma-glutamyl transferase increased, blood glucose increased, hepatic enzyme increased, aspartate aminotransferase increased, electrocardiogram qt prolonged, glucose urine present, anorexia, hyperglycemia musculoskeletal, posture abnormal, myalgia, back pain, buttock pain, muscular weakness, neck pain, musculoskeletal chest pain , coordination abnormal, dystonia, tardive dyskinesia, drooling, paresthesia, dizziness postural, convulsion, akinesia, hypokinesia, dysarthria, insomnia, agitation, anxiety, sleep disorder, depression, initial insomnia, libido decreased, nervousness, urinary incontinence, galactorrhea, oligomenorrhea, erectile dysfunction, sexual dysfunction, ejaculation disorder, gynecomastia, breast discomfort, nasal congestion, pharyngolaryngeal pain, dyspnea, rhinorrhea, rash, eczema, pruritus generalized, pruritus, hypotension, orthostatic hypotension, gait abnormal, upper respiratory tract infection, weight increased, decreased appetite, increased appetite, musculoskeletal, connective tissue disorders arthralgia, tremor, parkinsonism, dyskinesia, sedation, disturbance in attention, breast disorders, cough, hypertension | tardive dyskinesia (elder women), thrombotic thrombocytopenic purpura, pituitary gland adenomas, impaired fertility, menstrual disorder, breast cancer, disorder, pain, vaginal inflammation, dryness, haemorrhage, unintended pregnancy, menstruation delayed, menstrual disorder, amenorrhea | acute dystonia, erectile dysfunction, gynecomastia | 5-HT1a | Antagonist | (61-63) |
|  |  |  |  |  | 5-HT1c |  |  |
|  |  |  |  |  | 5-HT1d |  |  |
|  |  |  |  |  | 5-HT2a |  |  |
|  |  |  |  |  | 5-HT7 |  |  |
| **Ziprasidone** | Bipolar, schizophrenia | gynecomastia breast disorder, hyperprolactinemia, serotonin syndrome sexual dysfunction, anorexia, somnolence, extrapyramidal symptoms, akathisia, dizziness, respiratory tract infection, rhinitis, cough increased, rash, fungal dermatitis, abnormal vision, hypertonia, dystonia, dyskinesia, hypokinesia, tremor, paralysis and twitching, ECG changes, nocturia, oliguria, abdominal pain, flu syndrome, fever, accidental fall, face or tongue edema, chills, photosensitivity reaction, flank pain, hypothermia, motor vehicle accident tachycardia, hypertension, postural hypotension bradycardia, angina pectoris, atrial fibrillation first degree av block, bundle branch block, phlebitis, pulmonary embolus, cardiomegaly, cerebral infarct, cerebrovascular accident, deep, myocarditis, thrombophlebitis, vomiting, rectal haemorrhage, dysphagia, gum haemorrhage, jaundice, fecal impaction, gamma glutamyl transpeptidase increased, hematemesis, cholestatic jaundice, hepatitis, hepatomegaly, leukoplakia of mouth, fatty liver deposit, melena, hypothyroidism, hyperthyroidism, thyroiditis, anemia, ecchymosis, leukocytosis, leukopenia, eosinophilia, lymphadenopathy, thrombocytopenia, hypochromic anemia, lymphocytosis, monocytosis, basophilia, lymphedema, polycythemia, thrombocythemia, thirst, transaminase increased, peripheral edema, hyperglycemia, creatine phosphokinase increased, alkaline phosphatase increased, hypercholesteremia, dehydration, lactic dehydrogenase increased, albuminuria, hypokalemia, bun increased, creatinine increased, hyperlipemia, hypocholesteremia, hyperkalemia, hypochloremia, hypoglycemia, hyponatremia, hypoproteinemia, glucose tolerance decreased, gout, hyperchloremia, hyperuricemia, hypocalcemia, hypoglycemic reaction, hypomagnesemia, ketosis, respiratory alkalosis, myalgia, tenosynovitis, myopathy, agitation, extrapyramidal syndrome, tremor, dystonia, hypertonia, dyskinesia, hostility, twitching, paresthesia, confusion, vertigo, hypokinesia, hyperkinesia, abnormal gait, oculogyric crisis, hypesthesia, ataxia, amnesia, cogwheel rigidity, delirium, hypotonia, akinesia, dysarthria, withdrawal syndrome, buccoglossal syndrome, choreoathetosis, diplopia, incoordination, neuropathy, paralysis, myoclonus, nystagmus, torticollis, circumoral paresthesia, opisthotonos, reflexes increased, trismus dyspnea, pneumonia, epistaxis, hemoptysis, laryngismus, maculopapular rash, urticaria, alopecia, eczema, exfoliative dermatitis, contact dermatitis, vesiculobullous rash fungal dermatitis, conjunctivitis, dry eyes, tinnitus, blepharitis, cataract, photophobia, impotence, hematuria, polyuria, urinary retention, anorgasmia, glycosuria | menorrhagia, metrorrhagia, female lactation, vaginal haemorrhage, sexual dysfunction, uterine haemorrhage, menorrhagia, female lactation, amenorrhea, metrorrhagia | erectile dysfunction, abnormal ejaculation, male sexual dysfunction | 5-HT1a | Agonist | (62, 63) |
|  |  |  |  |  | 5-HT1b |  |  |
|  |  |  |  |  | 5-HT1d |  |  |
|  |  |  |  |  | 5-HT1e |  |  |
|  |  |  |  |  | 5-HT2a |  |  |
|  |  |  |  |  | 5-HT2c |  |  |
|  |  |  |  |  | 5-HT6 |  |  |
|  |  |  |  |  | 5-HT5a |  |  |
|  |  |  |  |  | 5-HT7 |  |  |
| **Citalopram** | Anti-depressant | loss of appetite, libido decreased, anxiety, easy bruising/bleeding, abnormal orgasm, insomnia, tremor, serotonin syndrome, dysphoric mood, irritability, agitation, dizziness, sensory disturbances (paresthesias), confusion, lethargy, emotional lability, insomnia, hypomania, ecchymoses, hematomas, epistaxis, petechiae to life-threatening haemorrhages, hyponatremia, hyponatremia include headache, difficulty concentrating, memory impairment, deficiency, unsteadiness, activation of mania/hypomania, seizures, impair judgment, thinking, or motor skills, mutagenesis, impaired fertility, teratogenic in pregnant women | breast pain, enlargement, discharge, endocrine disorder, amenorrhea, galactorrhea, menstrual disorder, vaginal discharge, infection increase, dryness, inflammation, haemorrhage, pain, discharge, unintended pregnancy | testicular disorder, testicular pain   ejaculation disorder | 5-HT2a | Antagonist | (63, 64) |
| **Imipramine Pamoate** | Anti-Depressant | constipation, hyperhidrosis, sinus, tremor, libido disorder, clinical worsening, impotence, orthostatic hypotension, hypertension, tachycardia, palpitation, myocardial infarction, arrhythmias, heart block, ECG changes, precipitation of congestive heart failure, stroke, confusional states (especially in the elderly), hallucinations, disorientation, delusions, anxiety, restlessness, agitation, insomnia, nightmares, hypomania, exacerbation of psychosis, numbness, tingling, paresthesias of extremities, incoordination, ataxia, tremors, peripheral neuropathy, extrapyramidal symptoms, seizures, alterations in EEG patterns, tinnitus, dry mouth, sublingual adenitis, blurred vision, disturbances of accommodation, mydriasis, paralytic ileus, urinary retention, delayed micturition, dilation of the urinary tract, skin rash, petechiae, urticaria, itching, photosensitization, edema (general or of face, tongue), drug fever, cross-sensitivity with desipramine, bone marrow depression including agranulocytosis, eosinophilia, purpura, thrombocytopenia, nausea, vomiting, anorexia, epigastric distress, diarrhea, peculiar taste, stomatitis, abdominal cramps, black tongue, increased or decreased libido, impotence, elevation or depression of blood sugar levels, inappropriate ADH secretion syndrome, jaundice (simulating obstructive), altered liver function, weight gain or loss, perspiration, flushing, increased urinary frequency, drowsiness, dizziness, weakness, fatigue, headache, parotid swelling, alopecia, proneness to falling | breast enlargement, galactorrhea | gynecomastia, ineffectiveness testicular swelling | 5-HT1a | Agonist | (61-63) |
|  |  |  |  |  | 5-HT2a | Antagonist |  |
|  |  |  |  |  | 5-HT2c |  |  |
|  |  |  |  |  | 5-HT7 |  |  |
|  |  |  |  |  | 5-HT6 | Partial agonist |  |
| **Nefazodone Hydrochloride** | Anti-Depressant | libido increase, serotonin syndrome, dry mouth, weakness, lightheadedness, yellowing of the skin or whites of eyes (jaundice), loss of appetite, severe nausea, abdominal (lower stomach) pain, rash or hives, seizure (convulsion), fainting, psychomotor retardation, tremor, hypertonia, libido decreased, respiratory pharyngitis, cough increased, special senses, blurred vision, abnormal vision, taste perversion, visual field defect, urogenital urinary frequency, urinary tract infection, urinary retention, breast pain, constipation, somnolence, dizziness, confusion, tinnitus, dyspnea, bronchitis, asthma, pneumonia, laryngitis, voice alteration, epistaxis, hiccup, hyperventilation, yawn, eye pain, dry eye, ear pain, abnormality of accommodation, diplopia, conjunctivitis, mydriasis, keratoconjunctivitis, hyperacusis, photophobia, deafness, angle-closure glaucoma, night blindness, taste loss, impotence cystitis, urinary urgency, polyuria, breast enlargement, urinary incontinence, hematuria, nocturia, kidney calculus, anorgasmia, oliguria | vaginitis, breast pain, uterine haemorrhagea, uterine fibroids enlarged, menorrhagia, vaginal haemorrhagea, amenorrhea, metrorrhagia | galactorrhea, erectile dysfunction, gynecomastia | 5-HT1a | Antagonist | (61-63) |
|  |  |  |  |  | 5-HT2a |  |  |
|  |  |  |  |  | 5-HT2c |  |  |
| **Clomipramine** | OCD, MDD | libido disorder, galactorrhea, perineal pain, loss or gain in appetite, hyperthyroidism, rhinitis, UTI, vertigo, pruritus genital, somnolence, tremor, dizziness, headache, insomnia, libido change, nervousness, myoclonus, paresthesia, memory impairment, anxiety, twitching, impaired concentration, depression, hypertonia, sleep disorder, psychosomatic disorder, yawning, confusion, speech disorder, abnormal dreaming, agitation, migraine, depersonalization irritability, emotional lability, panic reaction, aggressive reaction paresis, increased sweating, rash, pruritus dermatitis, acne, dry skin, urticaria, abnormal skin odor, dry mouth, constipation, nausea, dyspepsia, diarrhea, anorexia, abdominal pain, vomiting, flatulence, tooth disorder, gastrointestinal disorder, dysphagia, esophagitis, eructation, ulcerative stomatitis, fatigue, weight increase, flushing, hot flushes, chest pain, fever, allergy, pain, local edema, chills, weight decrease, otitis media, asthenia, halitosis, postural hypotension, palpitation, tachycardia, syncope, pharyngitis, rhinitis, sinusitis coughing bronchospasm, epistaxis, dyspnea, laryngitis, micturition disorder, urinary tract infection, micturition frequency, urinary retention, dysuria, cystitis, dysmenorrhea, lactation (nonpuerperal), menstrual disorder, vaginitis, breast enlargement, breast pain, ejaculation failure impotence, abnormal vision, taste perversion, tinnitus, abnormal lacrimation, mydriasis, conjunctivitis, anisocoria, blepharospasm, ocular allergy, vestibular disorder, musculoskeletal myalgia, back pain, arthralgia, muscle weakness, hemic, lymphatic purpura, anemia, thirst | menstrual disorder, vaginal, uterine inflammation, ovarian cysts, uterine, vaginal heamorrhage, endometrial hyperplasia, uterine inflammation, amenorrhea, breast pain, breast enlargement, leukorrhea, vaginitis, dysmenorrhea | erectile dysfunction, prostatic disorder, testicular swelling, impotence | 5-HT2a | Antagonist | (64, 66, 70) |
|  |  |  |  |  | 5-HT2b |  |  |
|  |  |  |  |  | 5-HT2c |  |  |
| **Escitalopram** | MDD, GAD, OCD | headache, serotonin syndrome, insomnia, diarrhea, neuropathy peripheral, tremor, endocrine disorder, sexual dysfunction, libido decrease/ increase, menopausal symptoms, genital candidiasis blocked or runny nose (sinusitis), decreased or increased appetite, anxiety, restlessness, abnormal dreams, difficulties falling asleep, feeling sleepy, dizziness, yawning, tremors, prickling of the skin, constipation, vomiting, dry mouth, increased sweating, pain in muscle, joints (arthralgia, myalgia), sexual disturbances (delayed ejaculation, erectile dysfunction, decreased libido, women may experience difficulties achieving orgasm) fatigue, fever increased weight, nettle rash (urticaria), itching (pruritus), grinding one’s teeth, agitation, nervousness, panic attack, confusion, disturbed sleep, taste disturbance, fainting (syncope) - enlarged pupils (mydriasis), visual disturbance, ringing in the ears (tinnitus), loss of hair, excessive menstrual bleeding, irregular menstrual period, decreased weight, fast heart beat - swelling of the arms or legs, nosebleeds, aggression, depersonalization, hallucination, slow heartbeat | impair fertility, interference with fallopian tube motility, breast disorder, pain, enlargement, cancer risk, menstrual disorder, ovarian cyst, premenstrual disorder, vaginitis atrophic, vaginal discharge, infection increase, dryness, pain, heamorrhage | ejaculation disorder, testicular pain, gynecomastia | 5-HT1a | Antagonist | (62-64) |
|  |  |  |  |  | 5-HT2a |  |  |
|  |  |  |  |  | 5-HT2c |  |  |
| **Paroxetine** | MDD, Panic disorder, OCD, social phobia, GAD, PMDD, vasomotor, menopausal symptoms | genital disorder, sexual dysfunction, libido decrease / increase, serotonin syndrome, impaired fertility, suicidal thoughts, behaviors, abnormal bleeding, hyponatremia, seizures, akathisia, angle closure glaucoma, cognitive, motor impairment, nausea, vomiting, fatigue, lethargy, headache, idiopathic thrombocytopenic purpura, events related to impaired hematopoiesis (including aplastic anemia, pancytopenia, bone marrow aplasia, agranulocytosis), atrial fibrillation, pulmonary edema, ventricular fibrillation, ventricular tachycardia (including torsades de pointes), pancreatitis, pancreatitis heamorrhagic, death, drug withdrawal syndrome, malaise, drug-induced liver injury, hepatic failure, jaundice, anaphylactoid reaction, angioedema, toxic epidermal necrolysis, liver necrosis, severe liver dysfunction, death, diabetes mellitus inadequate control, type 2 diabetes mellitus, neuroleptic malignant syndrome, paresthesia, somnolence, tremor, aggression, agitation, anxiety, confusional state, depression, disorientation, homicidal ideation, insomnia, restlessness, respiratory, pulmonary hypertension, skin, subcutaneous, hyperhidrosis, stevens-johnson syndrome | abdominal  pain, attention disturbance, suicidal ideation, bone fractures, agitation, impaired fertility, interference with fallopian tube motility, menstrual disorder, breast pain, ovarian cyst, vaginal infection increase, inflammation, uterine spasm, uterine sertraline enlarged, endometrial disorder | erectile dysfunction testicular pain, breast atrophy, endometrial disorder, gynecomastia | 5-HT2a | Agonist | (61-63) |
|  |  |  |  |  | 5-HT2b |  |  |
| **Nortriptyline** | MDD | hypotension, hypertension, tachycardia, palpitation, myocardial infarction, arrhythmias, heart block, stroke, confusional states (especially in the elderly) with hallucinations, disorientation, delusions, anxiety, restlessness, agitation, insomnia, panic, nightmares, hypomania, exacerbation of psychosis, numbness, tingling, paresthesias of extremities, incoordination, ataxia, tremors, peripheral neuropathy, extrapyramidal symptoms, seizures, alteration in EEG patterns, tinnitus, dry mouth, associated sublingual adenitis, blurred vision, disturbance of accommodation, mydriasis, constipation, paralytic ileus, urinary retention, delayed micturition, dilation of the urinary tract allergic skin rash, petechiae, urticaria, itching, photosensitization (avoid excessive exposure to sunlight), edema (general or of face, tongue), drug fever, cross-sensitivity with other tricyclic drugs, bone marrow depression, including agranulocytosis, eosinophilia, purpura, thrombocytopenia, nausea, vomiting, anorexia, epigastric distress, diarrhea, peculiar taste, page 8 of 12 stomatitis, abdominal cramps, black tongue, gynecomastia in the male, breast enlargement, increased or decreased libido, impotence, testicular swelling, elevation or depression of blood sugar levels, syndrome of inappropriate ADH secretion, jaundice (simulating obstructive), altered liver function, weight gain or loss, perspiration, flushing, urinary frequency, nocturia, drowsiness, dizziness, weakness, fatigue, headache, parotid swelling, alopecia, | galactorrhea, breast enlargement | erectile dysfunction | 5-HT1a | antagonist | (62) |
|  |  |  |  |  | 5-HT1c |  |  |
|  |  |  |  |  | 5-HT2a |  |  |
|  |  |  |  |  | 5-HT2b |  |  |
|  |  |  |  |  | 5-HT2c |  |  |
| **Desipramine** | MDD | tremors, seizures, tinnitus, blurred vision ,anorexia, vomiting, hepatitis, diarrhea, decrease or increase libido, hypotension, hypertension, palpitations, heart block, myocardial infarction, stroke, arrhythmias, premature ventricular contractions, ventricular tachycardia, ventricular fibrillation, sudden death, confusional states (especially in the elderly) with hallucinations, disorientation, delusions, anxiety, restlessness, agitation, insomnia, nightmares, hypomania, exacerbation of psychosis, numbness, tingling, paresthesias of extremities, incoordination, ataxia, peripheral neuropathy, extrapyramidal symptoms, NMS dry mouth, sublingual adenitis, disturbance of accommodation, mydriasis, increased intraocular pressure, constipation, paralytic ileus, urinary retention, delayed micturition, dilation of urinary tract, skin rash, petechiae, urticaria, itching, photosensitization (avoid excessive exposure to sunlight), edema (of face, tongue or general), drug fever, cross-sensitivity with other tricyclic drugs, bone marrow depressions including agranulocytosis, eosinophilia, purpura, thrombocytopenia, anorexia, nausea, vomiting, epigastric distress, peculiar taste, abdominal cramps, diarrhea, stomatitis, black tongue, hepatitis, jaundice (simulating obstructive), altered liver function, elevated liver function tests, increased pancreatic enzymes, gynecomastia in the male, breast enlargement, increased or decreased libido, impotence, painful ejaculation, testicular swelling, elevation or depression of blood sugar levels, syndrome of inappropriate ADH secretion, weight gain or loss, perspiration, flushing, urinary frequency, nocturia, parotid swelling, drowsiness, dizziness, proneness to falling, weakness, fatigue, headache, fever, alopecia, elevated alkaline phosphatase | menstrual disorder, galactorrhea, breast enlargement | gynecomastia, erectile dysfunction | 5-HT1a | Partial antagonist, agonist | (61) |
|  |  |  |  |  | 5-HT2a | Antagonist |  |
|  |  |  |  |  | 5-HT2c | Partial antagonist, agonist |  |
| **Trazodone Hydrochloride** | MDD | skin condition/edema, blurred vision, constipation, dry mouth, hypertension, hypotension, syncope, confusion, decreased concentration, disorientation, dizziness/light-headedness, drowsiness, fatigue, headache, nervousness, abdominal/gastric disorder, diarrhea, nausea/vomiting, aches/pains, neurological incoordination, tremors, eyes red/tired/itching, head full-heavy, malaise, nasal/sinus congestion, weight gain/ loss, hemolytic anemia, leukocytosis, cardiospasm, congestive heart failure, conduction block, orthostatic hypotension, syncope, palpitations, bradycardia, atrial fibrillation, myocardial infarction, cardiac arrest, arrhythmia, ventricular ectopic activity, including ventricular tachycardia, qt prolongation, prolonged qt interval, torsade de pointes, inappropriate ADH syndrome, diplopia, increased salivation, chills, unexplained death, weakness, cholestasis, jaundice, hyperbilirubinemia, liver enzyme alterations, increased amylase, methemoglobinemia, aphasia, ataxia, cerebrovascular accident, extrapyramidal symptoms, grand mal seizures, paresthesia, tardive dyskinesia, vertigo, abnormal dreams, agitation, anxiety, hallucinations, insomnia, paranoid reaction, psychosis, stupor, urinary incontinence, urinary retention, apnea, alopecia, hirsutism, leukonychia, pruritus, psoriasis, rash, urticaria, vasodilation, breast enlargement or engorgement, lactation | lactation, clitorism | priapism | 5-HT1a | Antagonist | (61, 62) |
|  |  |  |  |  | 5-HT1c |  |  |
|  |  |  |  |  | 5-HT2a | Antagonist, partial agonist |  |
|  |  |  |  |  | 5-HT2b |  |  |
|  |  |  |  |  | 5-HT2c |  |  |
| **Trimipramine Maleate** | MDD | libido decrease or increase, hypotension, hypertension, tachycardia, palpitation, myocardial infarction, arrhythmias, heart block, stroke, confusional states (especially the elderly) with hallucinations, disorientation, delusions, anxiety, restlessness, agitation, insomnia, nightmares, hypomania; exacerbation of psychosis, numbness, tingling, paresthesias of extremities, incoordination, ataxia, tremors, peripheral neuropathy, extrapyramidal symptoms, seizures, alterations in EEG patterns, tinnitus, syndrome of ADH secretion, dry mouth and, sublingual adenitis, blurred vision, disturbances of accommodation, mydriasis, constipation, paralytic ileus, urinary retention, delayed micturition, dilation of the urinary tract, skin rash, petechiae, urticaria, itching, photosensitization, edema of face and tongue, bone-marrow depression including agranulocytosis, eosinophilia, purpura, thrombocytopenia, fever, sore throat, pathological neutrophil depression, nausea, vomiting, anorexia, epigastric distress, diarrhea, peculiar taste, stomatitis, abdominal cramps, black tongue, elevation or depression of blood-sugar levels, jaundice (simulating obstructive), altered liver function, weight gain or loss, perspiration, flushing, urinary frequency, drowsiness, dizziness, weakness, fatigue, headache, parotid swelling, alopecia | galactorrhea, breast enlargement | gynecomastia, erectile dysfunction | 5-HT1a | Antagonist | (61) |
|  |  |  |  |  | 5-HT2c |  |  |
|  |  |  |  |  | 5-HT1d | Partial agonist, antagonist |  |
|  |  |  |  |  | 5-HT2a | Agonist |  |
| **Vortioxetine** | MDD | flatulence, dizziness, sexual dysfunction, constipation, vomiting, diarrhea, eye pain, serotonin syndrome, nausea, decrease libido, abnormal bleeding, activation of mania/hypomania, angle closure glaucoma, hyponatremia, dry mouth, pruritus, abnormal dreams, seizure, acute pancreatitis, generalized rash, ecchymosis, hematoma, epistaxis, petechiae to life-threatening heamorrhages, agitation, hallucinations, delirium coma, autonomic instability (tachycardia, labile blood, pressure, diaphoresis, flushing, hyperthermia), neuromuscular symptoms (tremor, rigidity, myoclonus, hyperreflexia, incoordination, suicide | sexual dysfunction | none reported | 5-HT1a | Agonist | (62) |
|  |  |  |  |  | 5-HT1b | Partial agonist |  |
|  |  |  |  |  | 5-HT7 | Antagonist |  |
| **Almotriptan Malate** | Migraine | breast disorder, pruritus, sweating, vertigo, nervous system disorder, nausea, dry mouth, paresthesia, headache, abdominal cramp or pain, asthenia, chills, back pain, chest pain, neck pain, fatigue, rigid neck, fever, photosensitivity reaction, vasodilation, palpitations, tachycardia, hypertension and syncope, diarrhea, vomiting, dyspepsia, gastroenteritis and increased thirst, colitis, gastritis, esophageal reflux, increased salivation, hyperglycemia, increased serum creatine phosphokinase, increased gamma glutamyl transpeptidase and hypercholesteremia, myalgia, arthralgia, arthritis, myopathy and muscle weakness, dizziness, somnolence, tremor, anxiety, hypesthesia, restlessness, CNS stimulation shakiness, change in dreams, impaired concentration, abnormal coordination, depressive symptoms, euphoria, hyperreflexia, hypertonia, nervousness, neuropathy, nightmares, nystagmus, insomnia, pharyngitis, rhinitis, dyspnea, laryngismus, sinusitis, bronchitis, hyperventilation, laryngitis, sneezing, epistaxis, diaphoresis, pruritus rash, dermatitis, erythema, ear pain, tinnitus, diplopia, dry eyes, eye pain, otitis media, parosmia, scotoma, conjunctivitis, eye irritation, hyperacusis, taste alteration, anaphylactic shock, confusional state, restlessness, hemiplegia, hypoesthesia, seizures, blepharospasm, vertigo acute myocardial infarction, coronary artery vasospasm, angina pectoris, tachycardia abdominal discomfort, abdominal pain, abdominal pain-upper, colitis, hypoesthesia-oral, swollen tongue, angioedema, cold sweat, erythema, hyperhidrosis, arthralgia, myalgia, pain in extremity, breast pain, malaise, peripheral coldness | dysmenorrhea | none reported | 5-HT1b | Agonist | (62, 63) |
|  |  |  |  |  | 5-HT1d |  |  |
| **Dihydroergotamine Mesylate** | Migraine | sore throat, nosebleeds, anxiety, rhinitis, muscle cramps, pharyngitis sinusitis, nausea vomiting diarrhea, altered sense of taste and smell, application site reaction, dizziness somnolence paraesthesia, hot flashes fatigue asthenia, mouth dry, stiffness, petechia, pruritus, rash, cold clammy skin, urticaria, herpes simplex, myalgia, muscular weakness, dystonia, arthralgia, rigidity, confusion, tremor, hypoesthesia, vertigo, speech disorder, hyperkinesia, stupor, abnormal gait, aggravated migraine, increased sweating, photophobia, conjunctivitis, abnormal lacrimation, abnormal vision, tinnitus, earache, eye pain, nervousness, euphoria, insomnia, concentration impaired, anorexia, depression, abdominal pain, dyspepsia, dysphagia, hiccup, increased salivation, esophagospasm, edema, palpitation, tachycardia, hypotension, peripheral ischeamia, angina, dyspnea, upper respiratory tract infections, bronchospasm, bronchitis, pleural pain, epistaxis, increased frequency of micturition, cystitis, feeling cold, malaise, rigors, fever, periorbital edema, flulike symptoms, shock, loss of voice, yawning, local anesthesia, increased blood pressure, serotonin syndrome peripheral vascular ischeamia, colonic ischeamia, cerebral heamorrhage, subarachnoid heamorrhage, stroke, pain, tightness, pressure in the chest or throat, neck and jaw | reproductive malfunction, pelvic inflammation, vaginitis, vaginal inflammation | none reported | 5-HT1a | Agonist | (61-63) |
|  |  |  |  |  | 5-HT1d |  |  |
|  |  |  |  |  | 5-HT2a |  |  |
|  |  |  |  |  | 5-HT2c |  |  |
| **Eletriptan Hydrobromide** | Migraine | breast pain, anesthesia, sweating, vertigo, diarrhea, tremor, urinary tract disorder, tinnitus, seizure, vomiting, dizziness, somnolence, headache, asthenia, paresthesia, flushing/feeling of warmth, chest tightness/pain/pressure, abdominal pain/discomfort/stomach pain/ cramps/pressure, dry mouth, dyspepsia, dysphagia throat tightness/difficulty swallowing, nausea, anaphylaxis, anaphylactoid hypersensitivity reactions, increase blood pressure, serotonin syndrome, peripheral vascular ischeamia, gastrointestinal vascular ischeamia, cerebral haemorrhage, subarachnoid heamorrhage stroke, arrhythmias, myocardial ischeamia, myocardial infarction prinzmetal’s angina | vaginal inflammation, menstrual disorder, menorrhagia | erectile dysfunction | 5-HT1a | Agonist | (62, 63) |
|  |  |  |  |  | 5-HT1b |  |  |
|  |  |  |  |  | 5-HT1d |  |  |
|  |  |  |  |  | 5-HT1f |  |  |
| **Frovatriptan** | Migraine | rhinitis, constipation, headache, dizziness, vomiting , abnormal vision, tinnitus, sweating, paresthesia, mouth dry dyspepsia, fatigue, chest pain, skeletal pain, flushing dysesthesia, hypoesthesia, tremor, hyperesthesia, migraine aggravated, involuntary muscle contractions, vertigo, ataxia, abnormal gait, speech disorder, hypertonia, hypotonia, abnormal reflexes, tongue paralysis, abdominal pain, diarrhea, dysphagia, flatulence, anorexia, esophagospasm, saliva increased, change in bowel habits, cheilitis, eructation, gastroesophageal reflux, hiccup, peptic ulcer, salivary gland pain, stomatitis, toothache, pain, asthenia, rigors, fever, hot flushes, malaise feeling of relaxation, leg pain, edema mouth, insomnia, anxiety, confusion, nervousness, agitation, euphoria, impaired concentration, depression, emotional lability, amnesia, thinking abnormal, depersonalization, abnormal dreaming, personality disorder, myalgia, back pain, arthralgia, arthrosis, leg cramps, muscle weakness, sinusitis, rhinitis, pharyngitis, dyspnea, hyperventilation, laryngitis, vision abnormal, eye pain, conjunctivitis, abnormal lacrimation, sweating increased, pruritis,, bullous eruption, tinnitus, ear ache,, hyperacusis, palpitation, tachycardia, bradycardia, thirst, dehydration, hypocalcemia, hypoglycemia, taste perversion, micturition frequency, polyuria, nocturia, renal pain, abnormal urine, abnormal ECG, platelet, epistaxis, purpura, autonomic nervous system syncope | none reported | none reported | 5-HT1b | Agonist | (62) |
|  |  |  |  |  | 5-HT1d |  |  |
| **Methysergide** | Migraine | rash, urinary problems, leg, chest pain, dizziness, vomiting, epigastric pain, psychic reactions, peripheral arterial insufficiency, peripheral oedema, pleural/pulmonary fibrosis, retroperitoneal fibrosis (RPF), valvular heart disease, drowsiness, nausea, increased appetite, weight gain, depression, muscle spasms, gastrointestinal disturbances, insomnia | vasoconstrictive phenomena, hair loss, ineffectiveness | mild temporal lobe disturbance | 5-HT1a | Agonist | (61-63, 71) |
|  |  |  |  |  | 5-HT2a | Antagonist |  |
|  |  |  |  |  | 5-HT2b |  |  |
|  |  |  |  |  | 5-HT2c |  |  |
|  |  |  |  |  | 5-HT7 |  |  |
| **Naratriptan** | Migraine | breast discharge, increase infections, vertigo, anxiety, sweating, hypothyroidism, paresthesia, serotonin syndrome, hypersensitive reactions, dizziness, fatigue, myocardial ischeamia, coronary artery vasospasm, peripheral vascular ischeamia, colonic ischeamia, abdominal pain, bloody diarrhea, high blood pressure, hypersensitivity, chest discomfort, carcinogenesis, mutagenesis, impaired fertility, nausea, drowsiness, malaise, neck/throat symptoms, body temperature changes, burning/stinging sensations,tachyarrhythmias, abnormal ECG, syncope, sinusitis, upper respiratory inflammation, tinnitus, allergic rhinitis, labyrinthitis, ears, nose, throat heamorrhage, hearing difficulty, dehydration, hyperlipidemia, hypercholesterolemia, hyperglycemia, glycosuria, ketonuria, vomiting, gastrointestinal discomfort, gastroenteritis, constipation, abnormal liver function, abnormal bilirubin levels, heamorrhoids, gastritis, esophagitis, salivary gland inflammation, oral itching, irritation, regurgitation, reflux, gastric ulcers, increased white bells, thrombocytopenia, heamoglobin defects, anemia, purpura, bronchitis, cough, pneumonia, tracheitis, asthma, pleuritis, airway constrictions, muscle pain, arthralgia, articular rheumatism, muscle cramps, spasms, joint, muscle stiffness, tightness, rigidity, bone, skeletal pain, vertigo, tremors, cognitive function disorders, sleep disorders, disorders of equilibrium, nerve syndromes, confusion, sedation, hyperesthesia, coordination disorder, paralysis of cranial nerves, decreased consciousness, dreams, altered sense of taste, neuralgia, neuritis, aphasia, hpoesthesia, motor retardation, muscle twitching, fasciculation, psychomotor restlessness, convulsion, fever chills, edema, swelling, allergies, allergic reaction, spasms mobility disorder, anxiety, depressive disorder, detachment, aggression, hostility, agitation, hallucinations, panic, hyperactivity, breast inflammation, breast discharge, decrease libido, lumps in breasts, sweating, skin rash, purinitus, urticaria, skin erythema, dermatitis, dermatosis, hair loss, alopecia, pruritic skin rash, acne, folliculitis, allergic skin reactions, macular skin/rash, skin photosensitivity, photodermatitis, skin flakiness, dry skin, bladder inflammation, polyuria, diuresis, urinary tract heamorrhage, urinary urgency, pyelitis, urinary incontinence | vaginal inflammation, fallopian tube inflammation, endometrium disorder | none reported | 5-HT1a | Agonist | (62) |
|  |  |  |  |  | 5-HT1b |  |  |
|  |  |  |  |  | 5-HT1d |  |  |
|  |  |  |  |  | 5-HT1f |  |  |
| **Sumatriptan** | Migraine | serotonin syndrome, weight gain/loss, cerebral heamorrhage, subarachnoid heamorrhage, stroke, peripheral vascular ischeamia and colonic ischeamia, abdominal pain, bloody diarrhea, increase or decrease blood pressure, palpitations, arrhythmia, hypertension, hypotension, pallor, pulsating sensations tachycardia, angina, atherosclerosis, bradycardia, cerebral ischeamia, cerebrovascular lesion, heart block, peripheral cyanosis, thrombosis, transient myocardial ischeamia vasodilation, sinusitis, allergic rhinitis, ear, nose or throat heamorrhage, external otitis, sensitivity to noise, hearing disturbances and otalgia, feeling of fullness in the ear(s), thyrotropin stimulating hormone (TSH) levels, galactorrhea, hyperglycemia, hypoglycemia, hypothyroidism, polydipsia, endocrine sores, lumps masses, fluid disturbances, disorders of sclera, mydriasis, blindness, visual disturbance, eye edema and swelling, eye irritation and itching, accommodation disorders, external ocular, muscle disorders, eye heamorrhage, eye pain keratitis and conjunctivitis, constipation, dysphagia gastroesophageal reflux, gastrointestinal bleeding, hematemesis, melena, peptic ulcer, gastrointestinal pain, dyspeptic symptoms, dental pain, gastritis, gastroenteritis, hypersalivation, distention, oral itching and irritation, salivary gland swelling, swallowing disorders, anemia, myalgia, muscle cramps, tetany, muscle atrophy, weakness tiredness; arthralgia and articular rheumatitis, acquired, musculoskeletal deformity, muscle rigidity, musculoskeletal, inflammation, phonophobia and photophobia, confusion, depression, difficulty concentrating, disturbance of smell, dysarthria, euphoria, facial pain, heat, sensitivity, incoordination, lacrimation, monoplegia, sleep disturbance, shivering, syncope, tremor, aggressiveness, apathy, bradylogia, cluster headache, convulsions, decreased, appetite, drug abuse, dystonic reaction, facial paralysis, hallucinations, hunger, hyperesthesia, hysteria, increased alertness, memory disturbance, neuralgia, paralysis, personality change, phobia, radiculopathy, suicide, twitching, agitation, anxiety, depressive disorders, detachment, motor dysfunction, neurotic disorders, psychomotor disorders, taste disturbances, raised intracranial pressure, dyspnea, asthma, hiccoughs, breathing, disorders, cough bronchitis, sweating, erythema, pruritus, rash, tenderness, drying, nodules or wrinkling, eczema, seborrheic dermatitis, malignant breast neoplasm, breast swelling, cysts, lumps masses of breasts, hematuria, urinary frequency, bladder inflammation, micturition disorders, urethritis, urinary infections, fever, fluid retention, overdose, edema, hematoma, lymphadenopathy, speech disturbance, voice disturbances, contusions, prickling sensation, abdominal aortic aneurysm, abnormal pulse, phlebitis, Raynaud syndrome, ECG changes (sinus arrhythmia, non-sustained ventricular premature beats, isolated, junctional or atrial ectopic beats, delayed activation of the right ventricle), chest discomfort, dehydration, disorder/discomfort nasal cavity and sinuses, ear infection, meniere disease, throat discomfort, vision alterations, colitis, disturbance of liver function tests, flatulence/eructation, gallstones, intestinal obstruction, pancreatitis retching, difficulty in walking, hypersensitivity to various agents, jaw discomfort, miscellaneous laboratory abnormalities, face swelling, disorder of mouth and tongue (burning of tongue, numbness of tongue, dry mouth), arthritis, backache, intervertebral disc disorder, neck pain/stiffness, joint disturbances (pain, stiffness, swelling, ache), chills, diplegia, disturbance of emotions, sedation, globus, hystericus, intoxication, myoclonia, neoplasm of pituitary, relaxation, sensation of lightness, simultaneous hot and cold sensations, stinging sensations, stress, tickling sensations, transient, hemiplegia yawning, influenza, lower respiratory tract infection, skin eruption, herpes, renal calculus | endometriosis, breast discharge, tenderness, lactation, cysts, lumps, abnormal menstrual cycle, breast cancer, dysmenorrhea, intermenstrual bleeding, abortion, menstruation symptoms, abnormal menstrual cycle, inflammation of fallopian tubes, | none reported | 5-HT1a | Agonist | (61-63) |
|  |  |  |  |  | 5-HT1b |  |  |
|  |  |  |  |  | 5-HT1d |  |  |
|  |  |  |  |  | 5-HT1f |  |  |
|  |  |  |  |  | 5-HT5a |  |  |
|  |  |  |  |  | 5-HT7 |  |  |
| **Zolmitriptan** | Migraine | breast cancer risk, breast growth, serotonin syndrome paresthesia, asthenia, sweating, anxiety, arrhythmia, increased infection, insomnia, necrosis, diarrhea heamorrhagic, increase blood pressure, dry mouth, hypertension, syncope, allergic reactions, depression, amnesia, hallucinations, emotional lability, dyspepsia, nauseam dizziness somnolence, vertigo, peripheral vascular ischeamia, infarction (presenting with abdominal pain and bloody diarrhea), Raynaud’s syndrome, cerebral heamorrhage, subarachnoid heamorrhage stroke, chest, throat, neck and jaw pain/tightness/pressure, arrhythmias, myocardial ischeamia, myocardial infarction prinzmetal angina, polyuria, urinary frequency or urgency | vaginal inflammation, uterine disorder, uterine fibroids enlarged | none reported | 5-HT1a | Agonist | (62, 63) |
|  |  |  |  |  | 5-HT1b |  |  |
|  |  |  |  |  | 5-HT1d |  |  |
|  |  |  |  |  | 5-HT1e |  |  |
|  |  |  |  |  | 5-HT1f |  |  |
|  |  |  |  |  | 5-HT2a |  |  |
|  |  |  |  |  | 5-HT2b |  |  |
|  |  |  |  |  | 5-HT7 |  |  |
